# Neuroinflammation associates with antioxidant heme oxygenase-1 response throughout the brain in persons living with HIV

**DOI:** 10.1007/s13365-020-00902-8

**Published:** 2020-09-10

**Authors:** Analise L. Gruenewald, Yoelvis Garcia-Mesa, Alexander J Gill, Rolando Garza, Benjamin B. Gelman, Dennis L. Kolson

**Affiliations:** 1 Department of Neurology, Perelman School of Medicine, University of Pennsylvania, 280 Clinical Research Building, 415 Curie Blvd., Philadelphia, PA 19104 USA; 2 Department of Pathology, University of Texas Medical Branch, 301 University Blvd., Keiller 3.118A, Route 0609, Galveston, TX 77555 USA

**Keywords:** HIV, Heme oxygenase, Regional, Antioxidant response, HIV-associated neurocognitive impairment, Neuroinflammation

## Abstract

Previous studies showed that persons living with HIV (PLWH) demonstrate higher brain prefrontal cortex neuroinflammation and immunoproteasome expression compared to HIV-negative individuals; these associate positively with HIV levels. Lower expression of the antioxidant enzyme heme oxygenase 1 (HO-1) was observed in PLWH with HIV-associated neurocognitive impairment (HIV-NCI) compared to neurocognitively normal PLWH. We hypothesized that similar expression patterns occur throughout cortical, subcortical, and brainstem regions in PLWH, and that neuroinflammation and immunoproteasome expression associate with lower expression of neuronal markers. We analyzed autopsied brains (15 regions) from 9 PLWH without HIV-NCI and 7 matched HIV-negative individuals. Using Western blot and RT-qPCR, we quantified synaptic, inflammatory, immunoproteasome, endothelial, and antioxidant biomarkers, including HO-1 and its isoform heme oxygenase 2 (HO-2). In these PLWH without HIV-NCI, we observed higher expression of neuroinflammatory, endothelial, and immunoproteasome markers in multiple cortical and subcortical regions compared to HIV-negative individuals, suggesting a global brain inflammatory response to HIV. Several regions, including posterior cingulate cortex, globus pallidus, and cerebellum, showed a distinct pattern of higher type I interferon (IFN)-stimulated gene and immunoproteasome expression. PLWH without HIV-NCI also had (i) stable or higher HO-1 expression and positive associations between (ii) HO-1 and HIV levels (CSF, plasma) and (iii) HO-1 expression and neuroinflammation, in multiple cortical, subcortical, and brainstem regions. We observed no differences in synaptic marker expression, suggesting little, if any, associated neuronal injury. We speculate that this may reflect a neuroprotective effect of a concurrent HO-1 antioxidant response despite global neuroinflammation, which will require further investigation.

**Electronic supplementary material:**

The online version of this article (10.1007/s13365-020-00902-8) contains supplementary material, which is available to authorized users.

## Introduction

Persistent oxidative stress and inflammation within the brain of persons living with HIV (PLWH) are thought to contribute to development of HIV-associated neurocognitive impairment (HIV-NCI) (Boerwinkle and Ances 2019; Chen et al. 2014; Hong and Banks 2015; Saylor et al. 2016; Spudich 2016; Tavazzi et al. 2014). In the absence of HIV infection, such potentially pathological processes typically induce expression of antioxidant response genes, which execute cytoprotective responses to acute cellular injury in the brain. Among those most strongly linked to neuronal injury and recovery in various models is the antioxidant/anti-inflammatory enzyme, heme oxygenase (HO), which exists in two isoforms: heme oxygenase 1 (HO-1) and heme oxygenase 2 (HO-2) (Chang et al. 2003; Dore et al. 1999; Parfenova et al. 2006; Schipper 2004; Schipper and Song 2015; Wang et al. 2006). HO-1 is highly inducible by a variety of factors and predominates in non-neuronal cells, while HO-2 is considered to be constitutively expressed and only slowly inducible by a few factors. In the brain, HO-2 predominates in neurons. The protective effects of HO-1 and HO-2 are downstream of their selective catabolism of heme, a highly toxic, pro-oxidative factor. Heme is released as a byproduct of hemoglobin degradation or, in the context of cellular stress, from cellular metabolic enzymes for which it serves as a critical cofactor. HO-mediated heme catabolism produces the anti-inflammatory, cytoprotective metabolites carbon monoxide and bilirubin (Chen 2014; Chen et al. 2005; Dore et al. 2000; Wu et al. 2019). In the human brain, expression of HO-1 and HO-2 RNA is reportedly higher in cortical areas (frontal, temporal, occipital) compared to pons and cerebellum (Takahashi et al. 1996), which suggests that HO enzymatic cytoprotective capacity may vary among brain regions.

We previously demonstrated that decreased HO-1 protein expression in the prefrontal cortex of PLWH associates with HIV-NCI and encephalitis, and that this HO-1 reduction is associated with the level of HIV expression, type I interferon (IFN)-stimulated gene expression, and macrophage activation (Ambegaokar and Kolson 2014; Gill et al. 2014). Notably, we observed a marked discordance (negative association) between HO-1 protein and RNA expression, which suggests a post-transcriptional loss of HO-1 protein (Kovacsics et al. 2017). In in vitro studies, we linked HO-1 expression to dysregulation of glutamate release from glial cells, further suggesting a direct link between HO-1 deficiency and excitotoxic injury as a mechanism of HIV-induced neuronal injury in vivo (Ambegaokar and Kolson 2014; Gill et al. 2015). HO-1 protein reduction was also observed in the neostriatum, but not in the occipital cortex or cerebellum, which suggests regional brain variability in HO-1 expression in response to HIV infection. Upregulation of IFN-stimulated genes and endothelia-associated genes has also been reported in the neostriatum in PLWH, which suggests concurrent host antioxidant responses and neuroinflammatory responses that extend beyond the prefrontal cortex (Gelman et al. 2012a).

We addressed several potential mechanisms by which HO-1 expression might be reduced by HIV infection in PLWH. PLWH have increased immunoproteasome subunit expression in the prefrontal cortex that associates with HIV load, HIV-NCI, and HIV-encephalitis (Nguyen et al. 2010). We found evidence in vitro that degradation of HO-1, but not HO-2, is enhanced through IFN-induced immunoproteasome expression (Kovacsics et al. 2017), thus potentially explaining the observed discordance between HO-1 protein and RNA expression in the prefrontal cortex of PLWH. We also addressed the potential role for known regulatory variations in the HO-1 promoter region (a (GT)_*n*_ dinucleotide repeat polymorphism). We found significant associations between this polymorphism and type I IFN–stimulated genes and T lymphocyte activation within the prefrontal cortex of PLWH (Gill et al. 2018). In a separate clinical cohort study of PLWH, we observed modulating effects of the HO-1 (GT)_*n*_ promoter dinucleotide repeat on risk of HIV-NCI (Garza et al. 2020). These observations suggest a balance between transcriptional and post-translational mechanisms of HO-1 modulation that determine parenchymal HO-1 levels in the setting of neuroinflammation in the prefrontal cortex, and they also suggest a role for HO-1 expression in modulating risk of HIV-NCI.

In this study, we sought to determine whether neuroinflammation and a concurrent HO-1 antioxidant response in PLWH extend to additional cortical and deep brain regions, and whether those processes associate with synaptic integrity. We analyzed 15 brain regions in an autopsy cohort of PLWH without HIV-NCI (*n* = 9) and HIV-negative individuals (*n* = 7). We determined the expression of HO-1 and HO-2 and defined their associations with plasma and cerebrospinal fluid (CSF) HIV levels, markers of neuroinflammation, synaptic integrity, endothelial adhesion, and immunoproteasome expression. Similar to our prior observations in the prefrontal cortex of PLWH, we observed higher expression of markers of neuroinflammation, endothelial activation and adhesion, and immunoproteasome expression in multiple cortical, subcortical, and brainstem regions compared with those of HIV-negative individuals. Notably, the posterior cingulate cortex, globus pallidus, and cerebellum expressed similar neuroinflammatory patterns. We also observed (i) similar or increased HO-1 expression levels in all brain regions in PLWH compared with those in HIV-negative individuals, (ii) positive association between HO-1 expression and HIV levels in CSF and plasma, (iii) positive association between HO-1 and neuroinflammatory markers, and (iv) no difference in expression of synaptic markers.

## Materials and methods

### National NeuroAIDS Tissue Consortium brain autopsy cohort and viral loads

A cohort consisting of 9 PLWH and 7 HIV-negative individuals was selected from the National NeuroAIDS Tissue Consortium (NNTC) (Table 1). We selected individuals on the basis of several criteria: (i) the availability of brain tissue specimens from multiple brain regions; (ii) the absence of neurocognitive impairment attributable to HIV infection (i.e., HIV-NCI) and HIV encephalitis; and, if possible, (iii) homozygosity for either “short” (S/S) or “long” (L/L) HO-1 promoter region (GT)_*n*_ dinucleotide repeat allele genotype. The rationale for seeking individuals with either the S/S or L/L genotype is that *HMOX1* promoters with short HO-1 (GT)_*n*_ repeats express higher basal *HMOX1* gene transcriptional activity and inducibility (Chen et al. 2002; Hirai et al. 2003; Rueda et al. 2007; Seu et al. 2012), and short HO-1 (GT)_*n*_ repeat alleles associate with less brain inflammation and lower risk of HIV-NCI in PLWH (Gill et al. 2018). This could potentially allow us to identify differences in HO-1 protein expression in various brain regions on the basis of the individual’s promoter genotype in the presence and absence of HIV infection.

**Table 1:** Demographic and clinical data for PLWH cohort

	HIV−	HIV+	*p* value
Demographic data, mean ± SD			
Number of subjects	7	9	
Age at death	60 ± 15.7	51 ± 11.0	0.196^§^
Hours postmortem	18 ± 9.9	19 ± 8.0	0.850^§^
Sex, *N* (%)			
Male	5 (71)	8 (89)	0.37^†^
Female	2 (29)	1 (11)	
Race, *N* (%)			
White	5 (71)	6 (67)	0.84^†^
Black	2 (29)	3 (33)	
Other/unknown	0 (0)	0 (0)	
Ethnicity, *N* (%)			
Hispanic	0 (0)	0 (0)	
Not Hispanic	7 (100)	9 (100)	
Disease parameters, mean ± SD			
log plasma HIV (c/ml)	–	4.2 ± 1.5	
log CSF HIV (c/ml)	–	2.9 ± 0.7	
CD4+ lymphocytes/mm^3^	–	120 ± 148.2	
Neurocognitive impairment, *N* (%)			
HIV-NCI	–	0 (0)	
Neuropsychological impairment (other)	–	4 (44)	
Neurocognitive normal	–	5 (56)	
No neurocognitive data	–	0 (0)	
ART, *N* (%)			
ART-experienced	–	8 (89)	
ART-naïve	–	0 (0)	
Unknown ART	–	1 (11)	

Data are presented as mean ± standard deviation (SD) or as population percentages

*CSF* cerebrospinal fluid, *HIV-NCI* HIV-associated neurocognitive impairment, *ART* antiretroviral therapy

^§^Statistical analysis: Student’s *t* test

^†^Statistical analysis: chi-square test

Among the 9 PLWH studied, none had diagnosed HIV-NCI, according to the Frascati criteria (Antinori et al. 2007). Four were categorized as “Neuropsychological impairment-other (NPIO),” indicating impairment that can be attributed to another comorbid condition distinct from HIV infection or AIDS-related CNS disease (co-infection, tumor, metabolic disease, other acquired neurological disease). The remaining 5 PLWH were categorized as neurocognitively normal. Eight of the PLWH were antiretroviral therapy (ART)-experienced. PLWH and HIV-negative individuals (*n* = 7) did not differ significantly by age, sex, race, ethnicity, or postmortem autopsy interval. Blood (*n* = 9) and CSF (*n* = 6) samples were obtained within 6 months of death and on the same day that neurocognitive testing was completed. Plasma and CSF HIV RNA levels were determined with the Amplicor HIV-1 Monitor test (v1.1 through v1.5, Roche).

### Monocyte-derived macrophage culture and small interfering RNA transfection

Peripheral blood mononuclear cells (PBMC) were isolated from volunteer donors by Ficoll density gradient centrifugation, and monocytes were isolated by adherence to gelatin-coated flasks (Cross et al. 2011). Isolated monocytes were plated at 10.5 × 10^4^ cells/cm^3^ on CellBIND plates (Corning) and cultured in Dulbecco’s modified Eagle’s medium (DMEM) supplemented with 10% fetal bovine serum (Thermo Scientific), 10% horse serum (Invitrogen), 1% non-essential amino acids (Invitrogen), 2 mM glutamine (Invitrogen), and 50 U/ml penicillin/streptomycin at 37 °C, 6% CO_2_. Monocyte-derived macrophages (MDM) were cultured for 7 days in vitro and inspected for differentiation. Silencer^®^ Select siRNAs (Ambion) were transfected at a final concentration of 20 nM using Lipofectamine RNAiMax (Invitrogen).

### Brain tissue sampling

Fresh-frozen brain tissue samples from the frontal cortex (FC), temporal cortex (TC), occipital cortex (OC), motor cortex (MC), sensory cortex (SC), anterior cingulate cortex (ACC), posterior cingulate cortex (PCC), amygdala (AM), caudate nucleus/putamen (CN), globus pallidus (GP), pons (PN), midbrain (MB), medulla (MED), cerebellum (CB), frontal white matter (FWM), and spinal cord (SPC) (Supplementary Table 1) stored at − 80 °C were dissected on dry ice. Samples from each region were divided into two pieces (i.e., adjacent pieces) for analysis of protein and RNA expression, respectively.

### Western blot

Protein lysates were prepared from 100 mg of tissue homogenized by silica bead beating and sonication in 7 volumes of buffer (10 mM Tris-HCl (pH 7.8), 0.5 mM dithiothreitol (DTT), 5 mM MgCl_2_, 0.03% Triton X-100) containing Complete Protease Inhibitor Cocktail (Roche Applied Science) and PhosSTOP Phosphatase Inhibitor Cocktail (Roche). Protein concentration was quantified using the detergent compatible (DC) protein assay (Bio-Rad). Proteins were resolved by SDS-PAGE and transferred to polyvinylidene fluoride (PVDF) membranes. Membranes were incubated with primary antibody (Supplementary Table 2) overnight at 4 °C. For infrared fluorescent detection of protein expression, membranes were incubated with IRDye-conjugated secondary antibody (Supplementary Table 3) for 1 h at room temperature and scanned with the Odyssey CLx Infrared Imagine System (LI-COR Biosciences). Background-corrected signal intensity of protein bands was measured using Image Studio Lite software (LI-COR Biosciences).

### HO-1 promoter (GT)_*n*_ repeat genotyping

HO-1 (GT)_*n*_ repeat lengths were determined by PCR of the (GT)_*n*_ repeat region with a 6-FAM-labeled forward primer, followed by fragment size determination on a capillary sequencer. In a subset of samples, PCR products were run on a 2% agarose gel to assess amplification of the target sequence–predicted sizes (homozygotes, heterozygotes). All samples were run at least twice from independent PCR reactions to ensure accurate and reproducible sizing and were determined to be accurate within ± 1 GT repeat. In rare (< 4%) cases where identical GT repeat lengths were not obtained in duplicate, samples were run additional times to confirm accurate repeat lengths. We assigned HO-1 (GT)_*n*_ alleles as short (S, < 27), medium (M, 27–34), or long (L, > 34) (GT)_*n*_ repeats (Gill et al. 2018).

### In-gel digestion and mass spectrometry analysis and confirmation of HO-1 detection

Human brain lysates were resolved by SDS-PAGE, with an identical lysate added to two lanes of a symmetrically organized gel. One half of the gel was stained for protein with Coomassie blue, while the other half was transferred to PVDF membrane and probed for HO-1 as described above. Gel bands for digestion were chosen by exact size according to corresponding bands recognized by the primary antibody used to probe the PVDF membrane. Each gel band was excised and further cut into 1 mm cubes (Shevchenko et al. 1996). These were de-stained with 50% methanol/1.25% acetic acid, reduced with 5 mM DTT (Thermo), and alkylated with 20 mM iodoacetamide (Sigma). Gel pieces were then washed with 20 mM ammonium bicarbonate (Sigma) and dehydrated with acetonitrile (Fisher). Trypsin (Promega) (5 ng/ml in 20 mM ammonium bicarbonate) was added to the gel pieces, and proteolysis was allowed to proceed overnight at 37 °C. Peptides were extracted with 0.3% triflouroacetic acid (J.T. Baker), followed by 50% acetonitrile. Extracts were combined, and the volume was reduced by vacuum centrifugation.

Extracted samples were analyzed on a Q-Exactive HF mass spectrometer (Thermo Fisher Scientific, San Jose, CA) coupled with an UltiMate 3000 nano UPLC system and an Easy-Spray source. Peptides were separated by reversed-phase (RP) HPLC on Easy-Spray RSLC C18 2 μm × 75 μm id × 50 cm column at 50 C. Mobile phase A consisted of 0.1% formic acid and mobile phase B of 0.1% formic acid/acetonitrile. Peptides were eluted into the mass spectrometer at 210 nl/min with each RP-LC run comprising a 125-min gradient from 1 to 5% B in 15 min and from 5 to 45% B in 110 min. The mass spectrometer was set to repetitively scan m/z from 300 to 1400 (*R* = 240,000) followed by data-dependent MS/MS scans on the twenty most abundant ions, minimum AGC 1e4, dynamic exclusion with a repeat count of 1, and repeat duration of 30 s (*R* = 15,000). FTMS full scan AGC target value was 3e6, while MSn AGC was 1e5, respectively. MSn injection time was 160 ms; microscans were set at one. Rejection of unassigned and 1+,6–8 charge states was set.

Peptide and protein identification were performed with the Sequest search engine accessed through Proteome Discoverer 2.0 (Thermo Fisher Scientific) using human reference proteome database from UniProt (canonical and isoform proteins; downloaded on January 4, 2018). Carbamidomethyl of Cys was defined as a fixed modification. Oxidation of Met and acetylation of protein N-termini were set as variable modifications. Trypsin was selected as the digestion enzyme, and a maximum of two missed cleavages per peptide was allowed. The precursor mass tolerance was set at 10 ppm, and the MS/MS fragment mass tolerance was set at 0.02 DA. The minimum peptide length was set at 7 amino acids. The false discovery rate (FDR) for peptides and proteins was set at 1%. The rest of the parameters were kept as default. The search results were exported and visualized in Scaffold 4.8.4 (Proteome Software). Mass spectrometry analysis and data processing were performed with support of the Children’s Hospital of Philadelphia Proteomics Core.

### Real-time quantitative PCR

Total RNA was prepared from 50 mg of fresh-frozen brain tissue (adjacent to tissue section used for preparation of protein lysates) using the RNeasy Lipid Tissue Mini Kit. RNA purity and concentration were determined with NanoDrop One UV-Vis spectrophotometer (Thermo Fisher Scientific), and 1 μg of total RNA was reverse transcribed to single-stranded complementary DNA (cDNA) using the High Capacity RNA-to-cDNA Kit (Applied Biosciences). Relative RNA expression was determined by RT-qPCR using 25 ng cDNA, TaqMan Fast Universal Master Mix (Applied Biosciences), and TaqMan primer/probe sets (*HMOX1*: Hs01110250_m1; *GAPDH*: Hs02786624_g1; *ISG15*: Ha00192713_m1; *MX1*: Hs00182073_m1; Applied Biosystems) in 10 μl reaction volumes. Reactions were run in triplicate on a StepOnePlus (Thermo Fisher Scientific). *GAPDH* was used as the reference gene, and RNA expression was calculated for each sample relative to the average expression of that gene across all brain samples.

### Statistical analysis

All quantifications are expressed as mean ± standard error of the mean (SEM). Protein and RNA expression data were log-transformed. Differences in expression of markers within individual brain regions between HIV-negative individuals and PLWH were evaluated by Student’s unpaired *t* test. Differences in mean expression of markers throughout the brain between HIV-negative individuals and PLWH were analyzed by Student’s paired *t* test, whereby mean expression of a marker in a given region within HIV-negative individuals was paired with mean expression of that marker in the same region within PLWH. Linear trends were analyzed by Pearson’s correlation with line of best fit determined by linear regression. Statistical significance was defined as *p* < 0.05. All statistical tests were performed using GraphPad Prism (version 8).

## Results

### HO-1 expression in multiple brain regions associates with CSF and plasma HIV levels in PLWH

**Fig. 1:**
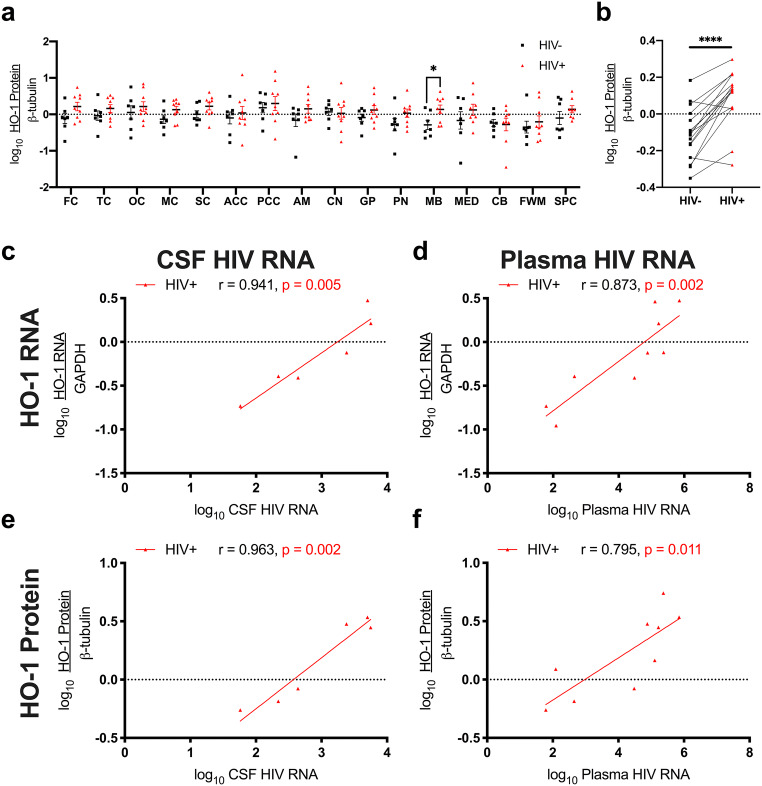
HO-1 protein (**a**, **b**, **e**, **f**) and RNA (**c**, **d**) were measured by Western blot and RT-qPCR, respectively. **a** Effect of HIV infection on HO-1 expression in individual brain regions was measured by Student’s unpaired *t* test. **b** Effect of HIV infection on whole-brain HO-1 expression was measured by Student’s paired *t* test, where the mean expression of HO-1 in each region in HIV-negative individuals was compared with the mean expression of HO-1 that region in PLWH. **c**, **e** CSF and **d**, **f** plasma HIV RNA were measured by RT-qPCR. Associations between frontal cortex HO-1 protein or RNA expression and plasma or CSF HIV RNA were analyzed by Pearson’s correlation with line of best fit by linear regression. *p < 0.05; ****p < 0.0001

We previously showed that HO-1 protein levels are lower in the prefrontal cortex and caudate of PLWH with HIV-NCI compared with those of HIV-negative individuals while HO-1 RNA levels are higher, suggesting an induction of HO-1 gene expression associated with post-translational HO-1 protein loss that may contribute to HIV-NCI (Gill et al. 2014). No such pattern was seen in the occipital cortex or cerebellum. In this study, we examined a broad distribution of brain regions (15 regions) and spinal cord in PLWH without HIV-NCI. We confirmed the relative specificity of the anti-HO-1 antibody used for our analyses (Supplementary Fig. 1). We analyzed human and transgenic mouse brain and spleen lysates (Supplementary Fig. 1a, c, d) and used mass spectrometry (Supplementary Fig. 1b) to confirm antibody specificity for HO-1 in human brain tissue. Comparing HO-1 protein expression within each brain region, we found higher HO-1 levels only in the midbrain in PLWH compared to HIV-negative individuals (Fig. 1a). However, the average of the means of HO-1 protein expression by region was significantly higher in PLWH, suggesting that increased HO-1 expression likely occurs in multiple brain regions (Fig. 1b). We also observed a positive association between HO-1 RNA and HO-1 protein expression in PLWH in 7 of 15 brain regions analyzed (temporal cortex, occipital cortex, motor cortex, sensory cortex, anterior cingulate cortex, posterior cingulate cortex, frontal white matter; Table 2).

**Table 2:** HO-1 protein associates positively with HO-1 RNA in PLWH

Region	HO-1 protein vs. HO-1 RNA			
	HIV-negative		PLWH	
	Pearson *r*	*p* value	Pearson *r*	*p* value
FC	0.301	0.563	0.586	0.097
TC	0.298	0.626	0.813	*0.008*
OC	0.612	0.144	0.673	*0.047*
MC	0.666	0.103	0.808	*0.008*
SC	0.150	0.776	0.686	*0.041*
ACC	− 0.009	0.985	0.667	*0.050*
PCC	0.470	0.287	0.675	*0.046*
AM	0.725	0.065	0.537	0.136
CN	0.240	0.605	0.577	0.104
GP	− 0.067	0.887	0.633	0.068
PN	0.192	0.679	0.534	0.139
MB	0.146	0.754	0.167	0.668
MED	0.162	0.728	0.525	0.182
CB	0.273	0.601	0.132	0.736
FWM	0.287	0.533	0.683	*0.043*
SPC	0.800	0.104	0.731	0.062

Correlations between HO-1 RNA and protein were analyzed by Pearson’s correlation. Italicized values: *p* < 0.05

Detection of CSF HIV RNA was successful in 6 of 9 PLWH while brain parenchymal HIV RNA was not detected in 8 of 9 PLWH, precluding a comprehensive analysis of HIV levels in the brain parenchyma. Nonetheless, CSF HIV RNA associated positively with brain HO-1 RNA, in 9 of 15 brain regions, including frontal cortex (Fig. 1c), five other cortical regions, amygdala, globus pallidus, and cerebellum (Table 3), similar to our prior observations in prefrontal cortex (Gill et al. 2014). We also found positive associations between plasma HIV RNA and HO-1 RNA in all brain regions except midbrain and frontal white matter (Fig. 1d, Table 3). CSF HIV RNA associated positively with HO-1 protein expression in 5 of 15 brain regions, including frontal cortex (Fig. 1e) and four other cortical regions (Table 3). Finally, we observed positive associations between plasma HIV RNA and HO-1 protein in 7 of 15 brain regions, including frontal cortex (Fig. 1f), five other cortical regions, and the pons (Table 3). These results confirm positive associations between plasma and CSF HIV expression and the host HO-1 response in multiple brain regions.

**Table 3:** Brain HO-1 RNA and protein associate with CSF and plasma HIV RNA in PLWH

Region	HO-1 RNA vs. CSF HIV RNA		HO-1 protein vs. CSF HIV RNA		HO-1 RNA vs. plasma HIV RNA		HO-1 protein vs. plasma HIV RNA	
	Pearson *r*	*p* value	Pearson *r*	*p* value	Pearson *r*	*p* value	Pearson *r*	*p* value
FC	0.941	*0.005*	0.963	*0.002*	0.873	*0.002*	0.795	*0.011*
TC	0.936	*0.006*	0.957	*0.003*	0.903	*0.001*	0.930	*0.000*
OC	0.766	0.076	0.843	*0.035*	0.822	*0.007*	0.670	*0.048*
MC	0.955	*0.003*	0.910	*0.012*	0.942	*0.000*	0.875	*0.002*
SC	0.969	*0.001*	0.764	0.077	0.876	*0.002*	0.759	*0.018*
ACC	0.988	*0.000*	0.935	*0.006*	0.866	*0.003*	0.634	0.067
PCC	0.782	0.066	0.791	0.061	0.741	*0.022*	0.709	*0.033*
AM	0.812	*0.050*	0.799	0.056	0.745	*0.021*	0.532	0.140
CN	0.540	0.269	0.779	0.068	0.650	0.058	0.506	0.164
GP	0.986	*0.000*	0.808	0.052	0.870	*0.002*	0.428	0.251
PN	0.792	0.061	0.770	0.073	0.730	*0.026*	0.770	*0.015*
MB	0.276	0.596	0.421	0.405	0.317	0.407	0.355	0.348
MED	0.804	0.101	0.845	0.071	0.750	*0.032*	0.663	0.073
CB	0.975	*0.001*	0.261	0.617	0.740	*0.023*	0.236	0.542
FWM	0.596	0.212	0.640	0.171	0.390	0.299	0.499	0.171
SPC	0.824	0.176	0.723	0.168	0.760	*0.048*	0.494	0.214

Correlations between HO-1 RNA or protein and CSF or HIV RNA in individual regions were analyzed by Pearson’s correlation. Italicized values: *p* < 0.05

### Expression of the constitutive HO-2 isoform is higher in frontal white matter in PLWH than in HIV-negative individuals

**Fig. 2:**
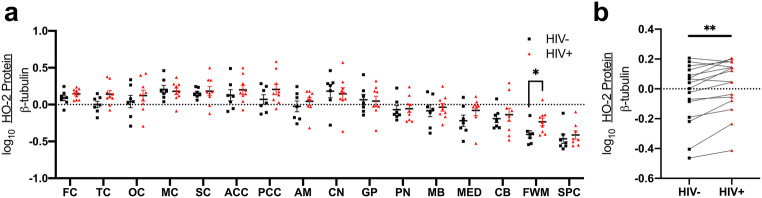
HO-2 protein was measured by Western blot. **a** Effect of HIV infection on HO-2 expression in individual brain regions was measured by Student’s unpaired *t* test. **b** Effect of HIV infection on whole-brain HO-2 expression was measured by Student’s paired *t* test, where the mean expression of HO-2 in each region in HIV-negative individuals was compared with the mean expression of HO-2 in that region in PLWH. *p < 0.05; **p < 0.01

The HO-2 isoform is considered to be constitutively expressed, but expression can change in response to certain stimuli (hypoxia, glucocorticoids) (Han et al. 2005; He et al. 2010; Vukomanovic et al. 2011; Zhang et al. 2006). In contrast to HO-1 expression in the brain, HO-2 is the predominant isoform expressed in neurons. We observed significantly higher HO-2 expression only in the frontal white matter of PLWH compared to HIV-negative individuals (Fig. 2a). However, similar to our HO-1 expression observation (Fig. 1b), when we compared the means of HO-2 expression for each region, we found higher HO-2 expression in PLWH (Fig. 2b).

### Immunoproteasome expression is higher in multiple brain regions in PLWH than in HIV-negative individuals

Our prior studies of PLWH showed increased immunoproteasome expression in the prefrontal cortex that associates with HIV-NCI and HIV encephalitis (Nguyen et al. 2010). We also showed a negative association between HO-1 protein and immunoproteasome expression in the prefrontal cortex in PLWH and evidence for immunoproteasome-associated degradation of HO-1 (Kovacsics et al. 2017). To determine whether this association is consistent throughout the brain in PLWH, we quantified expression of immunoproteasomes (LMP7 and Pa28α subunits) and constitutive proteasomes (β5 subunit) in additional brain regions.

**Fig. 3:**
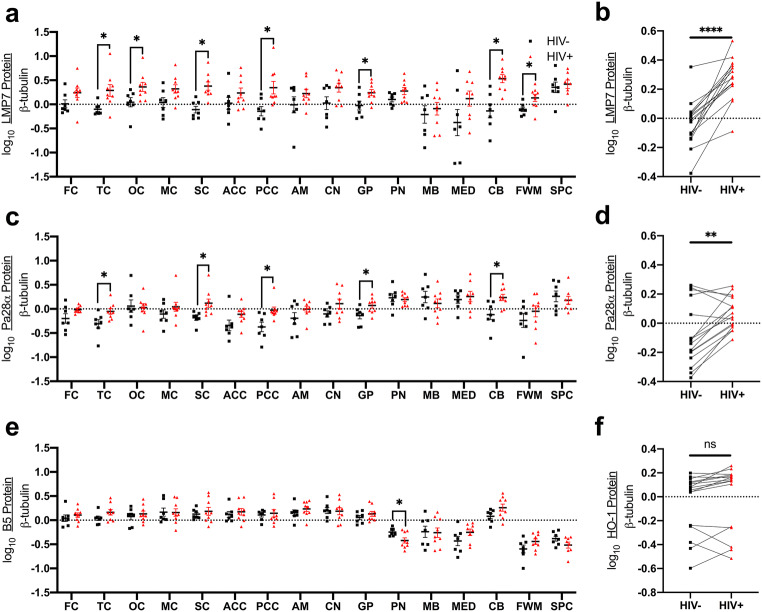
LMP7 (**a**, **b**), Pa28α (**c**, **d**), and B5 (**e**, **f**) protein expression levels were measured by Western blot. **a**, **c**, **e** Effect of HIV infection on proteasome subunit expression in individual brain regions was measured by Student’s unpaired *t* test. **b**, **d**, **f** Effect of HIV infection on whole-brain proteasome subunit expression was measured by Student’s paired *t* test. *p < 0.05; **p < 0.01; ****p < 0.0001

HIV infection associated with higher expression of immunoproteasome subunits in multiple cortical and subcortical regions (LMP7: temporal cortex, occipital cortex, sensory cortex, posterior cingulate cortex, globus pallidus, cerebellum, frontal white matter; Pa28α: temporal cortex, sensory cortex, posterior cingulate cortex, globus pallidus, cerebellum; Fig. 3a–d). These immunoproteasome associations stand in contrast to the lack of changes in constitutive proteasome expression (β5 subunit), in PLWH compared to HIV-negative individuals with the exception of lower β5 expression in the pons (Fig. 3e, f). We did not find a consistent relationship between HO-1 and LMP7 expression levels across brain regions in PLWH (Supplementary Table 4).

### HO-1 expression in multiple brain regions associates with type I IFN–stimulated genes in PLWH

**Fig. 4:**
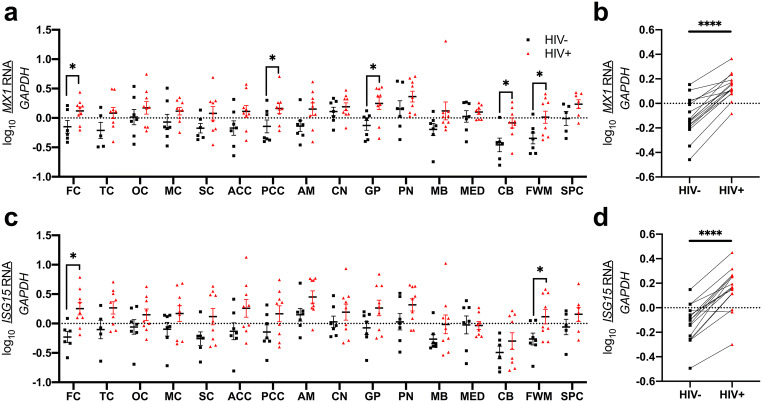
**a**, **b** *MX1* and **c**, **d** *ISG15* RNA expression levels were measured by RT-qPCR. **a**, **c** Effect of HIV infection on type I IFN–stimulated gene expression in individual brain regions was measured by Student’s unpaired *t* test. **b**, **d** Effect of HIV infection on whole-brain IFN-stimulated gene expression was measured by Student’s paired *t* test. *p < 0.05; ****p < 0.0001

We next determined the association between HIV infection, type I IFN–stimulated genes (*ISG15* and *MX1*), and HO-1 expression. We found increased type I IFN–stimulated gene expression in multiple cortical and subcortical regions (Fig. 4a–d). We observed higher *MX1* (Fig. 4a, b: frontal and posterior cingulate cortices, globus pallidus, cerebellum, and frontal white matter) and higher *ISG15* (Fig. 4c, d: frontal cortex and frontal white matter). We also identified several positive associations between *ISG15* and (i) HO-1 RNA (temporal cortex, anterior and posterior cingulate cortices, frontal white matter, Table 4) and (ii) HO-1 protein (anterior cingulate cortex, amygdala, frontal white matter, spinal cord; Table 4) in PLWH. *MX1* also associated positively with HO-1 RNA: temporal, occipital, anterior cingulate cortices, amygdala, cerebellum, frontal white matter, and spinal cord (Table 4) in PLWH. In contrast, in HIV-negative individuals, we did not find clear evidence of an association between HO-1 and *ISG15* or *MX1* (Supplementary Table 5).

**Table 4:** Associations between brain HO-1 and type I IFN–stimulated genes in PLWH

Region	*ISG15* RNA vs. HO-1 RNA		*ISG15* RNA vs. HO-1 protein		*MX1* RNA vs. HO-1 RNA		*MX1* RNA vs. HO-1 protein	
	Pearson *r*	*p* value	Pearson *r*	*p* value	Pearson *r*	*p* value	Pearson *r*	*p* value
FC	0.497	0.173	0.311	0.415	0.510	0.161	− 0.239	0.535
TC	0.712	*0.032*	0.605	0.085	0.706	*0.034*	0.435	0.241
OC	0.588	0.096	0.559	0.118	0.670	*0.048*	0.412	0.270
MC	0.589	0.096	0.588	0.096	0.355	0.348	0.347	0.361
SC	0.615	0.078	0.213	0.583	0.350	0.355	− 0.044	0.910
ACC	0.755	*0.019*	0.725	*0.027*	0.736	*0.024*	0.472	0.199
PCC	0.714	*0.031*	0.613	0.080	0.630	0.069	0.480	0.1915
AM	0.634	0.067	0.724	*0.027*	0.741	*0.022*	0.466	0.207
CN	0.204	0.599	0.556	0.120	0.481	0.190	0.590	0.094
GP	0.540	0.134	0.655	0.055	0.403	0.282	0.505	0.166
PN	0.247	0.522	0.203	0.600	0.107	0.783	− 0.153	0.695
MB	0.634	0.067	− 0.297	0.438	0.563	0.115	− 0.308	0.420
MED	0.489	0.219	0.191	0.650	0.000	> 0.9999	− 0.516	0.191
CB	0.559	0.117	− 0.216	0.577	0.750	*0.020*	0.128	0.744
FWM	0.738	*0.023*	0.802	*0.009*	0.768	*0.016*	0.650	0.058
SPC	0.680	0.093	0.839	*0.018*	0.798	*0.031*	0.583	0.170

Correlations between HO-1 RNA or protein and *ISG15* or *MX1* RNA in individual regions were analyzed by Pearson’s correlation. Italicized values: *p* < 0.05

### HO-1 expression in multiple brain regions associates with markers of endothelial activation and adhesion in PLWH

**Fig. 5:**
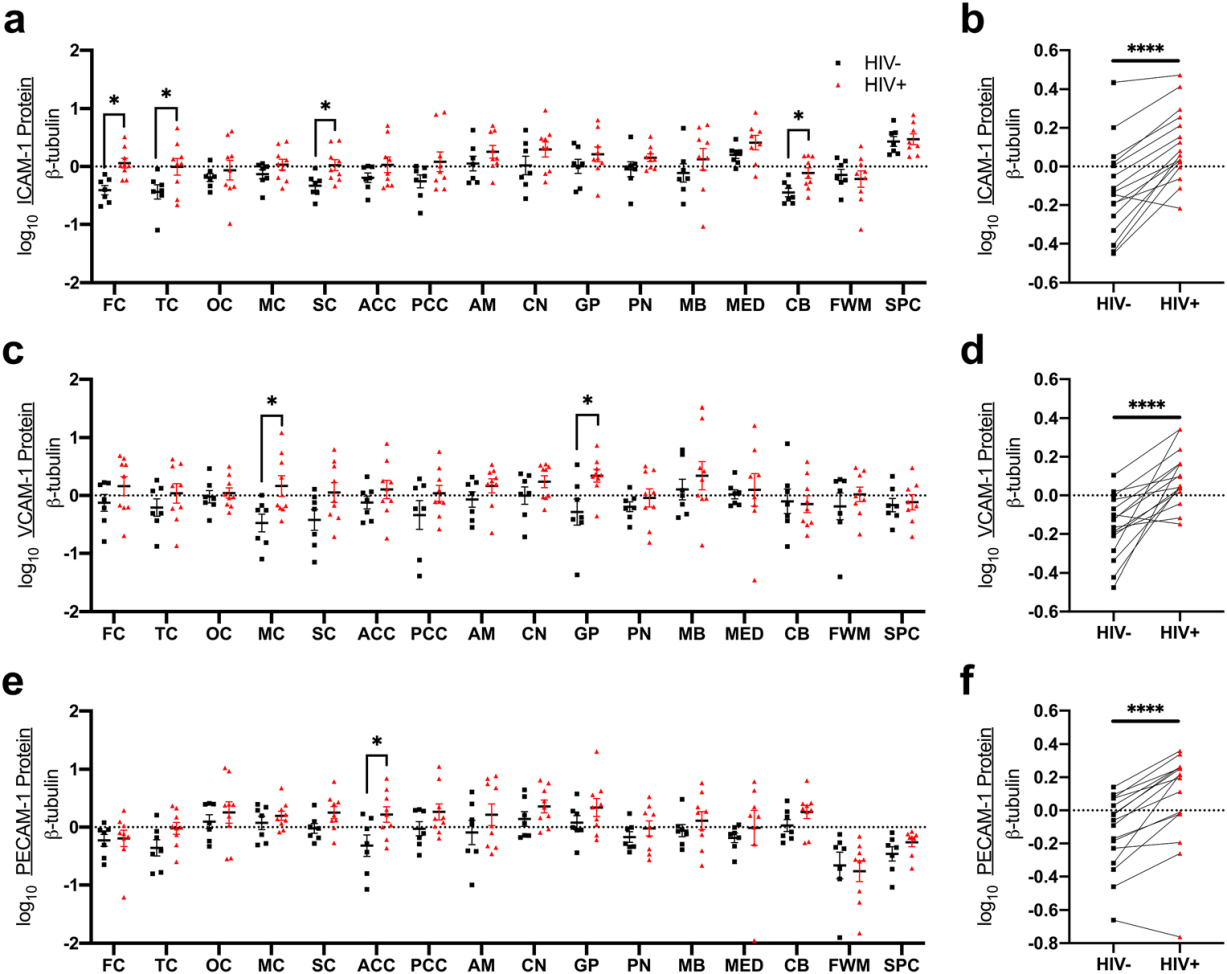
ICAM-1 (**a**, **b**), VCAM-1 (**c**, **d**), and PECAM-1 (**e**, **f**) protein expression levels were measured by Western blot. **a**, **c**, **e** Effect of HIV infection on endothelial adhesion molecule expression in individual brain regions was measured by Student’s unpaired *t* test. **b**, **d**, **f** Effect of HIV infection on whole-brain endothelial adhesion molecule expression was measured by Student’s paired *t* test. *p < 0.05; ****p < 0.0001

Dysfunction and/or injury to the neurovascular unit (defined as capillary endothelia, pericytes, astrocytic foot processes, neuronal processes, perivascular macrophages, along with the CNS extracellular space, subarachnoid space, and blood plasma) is thought to be a major contributor to the development of HIV-NCI, perhaps independent from brain neuroinflammation (Gelman 2015; Gelman et al. 2012a, 2018; Hill et al. 2014). We examined several endothelial adhesion molecules (ICAM-1, VCAM-1, PECAM-1) and observed higher expression of each overall, and in multiple cortical and subcortical regions in PLWH compared to HIV-negative individuals: (i) ICAM-1 (frontal, sensory, and temporal cortices and cerebellum; Fig. 5a, b), (ii) VCAM-1 (motor cortex and globus pallidus; Fig. 5c, d), and (iii) PECAM-1 (anterior cingulate cortex; Fig. 5e, f).

We determined the associations of these endothelial markers with HO-1 expression. VCAM-1 and PECAM-1, but not ICAM-1, associated positively with HO-1 protein expression in PLWH in multiple cortical, subcortical, and brainstem regions (VCAM-1: frontal, temporal, anterior cingulate cortices; pons; midbrain; and spinal cord; PECAM-1: temporal, occipital and posterior cingulate cortices and globus pallidus; Table 5). Each also associated positively with HO-1 RNA in multiple cortical, subcortical, and brainstem regions in PLWH (VCAM-1: amygdala and caudate; PECAM-1: six cortical regions, caudate, and pons) (Table 5). We did not find consistent associations between HO-1 and endothelial adhesion molecules in HIV-negative individuals (Supplementary Table 6).

**Table 5:** Associations between brain HO-1 and endothelial adhesion molecules in PLWH

Region	ICAM-1 protein vs. HO-1 RNA		ICAM-1 protein vs. HO-1 protein		VCAM-1 protein vs. HO-1 RNA		VCAM-1 protein vs. HO-1 protein		PECAM-1 protein vs. HO-1 RNA		PECAM-1 protein vs. HO-1 protein	
	Pearson *r*	*p* value	Pearson *r*	*p* value	Pearson *r*	*p* value	Pearson *r*	*p* value	Pearson *r*	*p* value	Pearson *r*	*p* value
FC	0.219	0.571	0.407	0.277	0.320	0.401	0.847	*0.004*	0.738	*0.023*	0.258	0.503
TC	0.049	0.900	0.180	0.644	0.385	0.306	0.827	*0.006*	0.870	*0.002*	0.752	*0.020*
OC	− 0.324	0.395	0.141	0.719	0.285	0.458	0.039	0.921	0.768	*0.016*	0.888	*0.001*
MC	0.279	0.468	0.114	0.771	0.611	0.081	0.626	0.071	0.753	*0.019*	0.513	0.158
SC	0.435	0.242	0.132	0.735	0.497	0.173	0.535	0.138	0.777	*0.014*	0.175	0.652
ACC	0.199	0.607	0.294	0.443	0.556	0.120	0.906	*0.001*	0.751	*0.020*	0.569	0.110
PCC	− 0.093	0.812	0.134	0.731	0.308	0.420	0.364	0.336	0.544	0.131	0.782	*0.013*
AM	− 0.051	0.895	− 0.275	0.474	0.807	*0.009*	0.439	0.238	0.571	0.108	0.659	0.054
CN	− 0.190	0.625	− 0.089	0.821	0.780	*0.013*	0.407	0.277	0.708	*0.033*	0.461	0.212
GP	0.161	0.679	0.125	0.748	0.615	0.078	0.619	0.076	0.623	0.073	0.679	*0.044*
PN	− 0.215	0.578	0.186	0.633	0.207	0.592	0.748	*0.021*	0.768	*0.016*	0.551	0.125
MB	− 0.039	0.920	0.293	0.481	0.243	0.528	0.778	*0.023*	0.299	0.435	0.562	0.147
MED	− 0.160	0.706	0.332	0.422	0.556	0.152	0.686	0.060	0.493	0.214	0.364	0.375
CB	− 0.401	0.285	− 0.203	0.601	0.253	0.512	− 0.065	0.869	0.497	0.174	− 0.004	0.992
FWM	0.399	0.288	0.094	0.810	0.364	0.336	0.504	0.167	0.377	0.317	0.226	0.558
SPC	0.483	0.273	0.352	0.393	0.315	0.491	0.859	*0.006*	0.719	0.069	0.532	0.175

Correlations between HO-1 RNA or protein and ICAM-1, VCAM-1, or PECAM-1 protein in individual regions were analyzed by Pearson’s correlation. Italicized values: *p* < 0.05

### Synaptic marker expression in multiple brain regions is similar in PLWH and in HIV-negative individuals

**Fig. 6:**
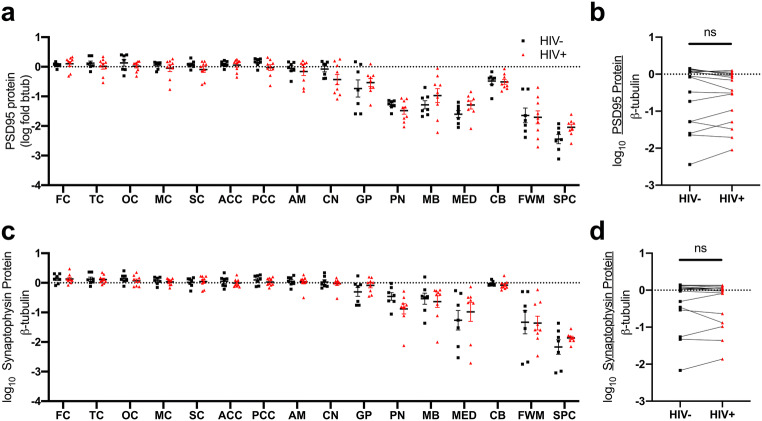
**a**, **b** PSD95 and **c**, **d** synaptophysin protein expression levels were measured by Western blot. **a**, **c** Effect of HIV infection on synaptic marker expression in individual brain regions was measured by Student’s unpaired *t* test. **b**, **d** Effect of HIV infection on synaptic marker expression was measured by Student’s paired *t* test

Because the induction of HO-1 expression is associated with neuroprotection in various injury response models including HIV infection (Gill et al. 2014, 2015), we measured markers of synaptic integrity in PLWH. To do this, we analyzed expression of presynaptic (synaptophysin) and postsynaptic (PSD95) markers. Expression of each was similar in PLWH compared to HIV-negative individuals (Fig. 6a–d) and neither associated with HO-1 protein or RNA expression in PLWH (Supplementary Tables 7 and 8).

### The HO-1 promoter region (GT)_*n*_ variant genotype does not associate with brain HO-1 expression

The HO-1 promoter region contains a (GT)_*n*_ dinucleotide repeat that modulates basal HO-1 expression and gene inducibility, specifically with shorter HO-1 (GT)_*n*_ repeats associating with higher basal HO-1 promoter activity (Chen et al. 2002; Hirai et al. 2003; Rueda et al. 2007; Seu et al. 2012), as well as better outcomes in inflammatory and oxidative stress-associated diseases (Bai et al. 2010; Chen et al. 2012; Rueda et al. 2007; Seu et al. 2012; Vázquez-Armenta et al. 2013). Short (S) (GT)_*n*_ repeat length alleles are defined as those with less than 27 (GT)_*n*_ repeats, while long (L) alleles are defined as those with more than 34 (GT)_*n*_ repeats (Gill et al. 2018; Kaartokallio et al. 2018; Vilander et al. 2019). Our current study cohort included 5 “SS” homozygous individuals (3 PLWH, 2 HIV-negative) and 4 “LL” homozygous individuals (3 PLWH, 1 HIV-negative). We did not find a significant difference in HO-1 protein or RNA expression in any individual brain region in PLWH based upon the HO-1 (GT)_*n*_ repeat allele genotype (Supplementary Fig. 2a–d).

## Discussion

Neuroinflammation and oxidative stress are considered central to the pathogenesis of neurocognitive impairment in PLWH, and analyses of associated biomarkers are typically limited to only a few brain regions. In previous studies of prefrontal cortex in PLWH compared to HIV-negative individuals, we and others observed increased expression of markers of neuroinflammation (IFN-stimulated genes, endothelial markers) and immunoproteasome expression that associate with HIV-NCI and HIV-encephalitis (Gelman et al. 2012a, 2012b; Gelman and Nguyen 2010; Nguyen et al. 2010). In the prefrontal cortex, we also observed reduced protein levels of the antioxidant/anti-inflammatory enzyme, HO-1 (Gill et al. 2014), despite increased levels of HO-1 RNA (Kovacsics et al. 2017). This discordant HO-1 protein and RNA expression associated with HIV load in the brain and CSF, neuroinflammation, immunoproteasome expression, and HIV-NCI. Notably, PLWH with HIV-NCI had significantly lower HO-1 expression in the prefrontal cortex compared to PLWH without HIV-NCI in the same cohort. In in vitro studies, we showed that HIV infection drives decreased HO-1 expression in monocyte-derived macrophages, and that immunoproteasome expression enhances HO-1 degradation (Gill et al. 2014; Kovacsics et al. 2017). We subsequently linked this reduced glial cell HO-1 expression with release of neurotoxic levels of glutamate, which has been associated with HIV-NCI (Ambegaokar and Kolson 2014; Cassol et al. 2014; Espey et al. 2002, 1999; Ferrarese et al. 2001; Gelman et al. 2012a; Peluso et al. 2013; Sailasuta et al. 2012; Saing et al. 2016; Tremolizzo et al. 2002). We concluded that HIV infection and HIV-driven neuroinflammation and immunoproteasome induction could lead to accelerated HO-1 protein degradation and loss, leading to neuronal injury and, ultimately, HIV-NCI. Finally, we also showed that the HO-1 gene promoter region (GT)_*n*_ dinucleotide repeat genetic variation, which is known to modulate HO-1 transcriptional activity, associates with a risk of HIV-NCI and neuroinflammation in PLWH in a pattern consistent with a role for HO-1 in reducing risk of HIV-NCI (Garza et al. 2020; Gill et al. 2018).

We thus hypothesized that PLWH have HIV-driven neuroinflammation and immunoproteasome expression throughout the brain that associates with HO-1 expression, and that this might associate with synaptic integrity in multiple regions, or even predominantly in selected regions. Based upon our prior observations in the prefrontal cortex comparing PLWH with and without HIV-NCI and abundant literature defining HO-1 induction in response to inflammation and oxidative stress, we hypothesized that this cohort of PLWH without HIV-NCI would have normal or even increased levels of brain HO-1 expression along with increased neuroinflammation and immunoproteasome expression. Our results indeed showed stable or increased HO-1 expression and increased neuroinflammation, endothelial marker expression, and immunoproteasome expression in multiple cortical, subcortical, and brainstem regions in PLWH compared to HIV-negative individuals (see schematic Supplementary Fig. 3). We felt that examining individuals with predictably distinct HO-1 (GT)_*n*_ genotypes might allow us to identify differences in regional brain HO-1 expression that might be influenced by the individual’s promoter genotype translational HO-1 expression capacity, in both the presence and absence of HIV infection. However, we could identify only 6 PLWH individuals of SS and LL allele genotypes who met all three inclusion criteria. Among the HIV-negative individuals, 3 had these genotypes, thus limiting the statistical powering of the analysis.

The observed stable or increased HO-1 expression in PLWH without HIV-NCI is in contrast to our previously reported decreased HO-1 expression in the prefrontal cortex in PLWH with HIV-NCI. These data suggest that although induction of pathological neuroinflammation occurs in multiple brain regions, induction of a protective HO-1 response also occurs in such regions in PLWH without HIV-NCI. These data also suggest that the HO-1 response is pathologically suppressed in PLWH who develop HIV-NCI, in part through immunoproteasome-mediated degradation of HO-1 (Kovacsics et al. 2017). Thus, in conjunction with our previous findings, this study is consistent with the hypothesis that therapeutic induction of HO-1 in PLWH may limit neuroinflammation and provide neuroprotection against HIV-NCI (see schematic Supplementary Fig. 3). We did not observe differences in synaptic marker expression in this cohort, which suggests that these concurrent inflammatory and antioxidant responses were not associated with synaptic neuronal injury in these individuals, albeit as determined only by the level of sensitivity of detection of differences in expression levels of synaptic proteins.

Our finding of increased immunoproteasome expression in these PLWH without HIV-NCI was not unexpected. Immunoproteasome expression is increased within the CNS often in response to viral infection (including HIV infection) (Jimenez-Guardeno et al. 2019; Nguyen et al. 2010), where the immunoproteasomes serve to degrade viral proteins as a host defense mechanism. The subunit Pa28α is the immunoproteasome regulatory subunit, and the subunit LMP7 is one of the two immunoproteasome catalytic subunits, all of which assemble to form the functional immunoproteasome (Pickering et al. 2010). Expression of each of the immunoproteasome subunits is enhanced by pro-inflammatory mediators associated with HIV infection, including TNF-α and IFN-γ (Basler et al. 2013). The constitutive proteasome, marked by expression of the B5 subunit, is constitutively expressed to serve functions of host cellular protein degradation to maintain cellular homeostasis (Nandi et al. 1997). Replacement of constitutive proteasomes by immunoproteasomes occurs under pathologic conditions, such as viral infections, through “substitution” of constitutive subunits by “immuno” subunits in the proteasome, thus generating the immunoproteasome for host defense against the invading pathogen (Fruh et al. 1994; Pickering et al. 2010). Together with our previous study of PLWH with HIV-NCI, we speculate that the reduction of HO-1 expression that associates with HIV-NCI occurs as a result of achieving a threshold level of immunoproteasome expression and associated degradative activity in those PLWH.

We also observed higher HO-2 expression in the frontal white matter in PLWH compared to uninfected individuals, which was unexpected, as HO-2 is considered to be constitutively expressed. One possibility is that changes in HO-2 expression directly reflect changes in axons, the major cellular subcomponent of brain white matter. Because HO-2 in the brain predominates in neurons while HO-1 predominates in glia and other non-neuronal cells and because recovery from neuronal injury is supported by HO-2 (Chen 2014; Chen et al. 2003), we speculate that the changes in HO-2 expression within the white matter might reflect changes in neuronal axons. We did not find evidence for loss of synaptic or axonal markers in the frontal white matter in PLWH in our cohort, and we cannot therefore conclude that the elevation of HO-2 that we observed represents a reparative response to neuronal injury. Nonetheless, chronic HIV is often associated with deep white matter injury, and we speculate that the higher HO-2 levels that we observed could indeed represent a response to undetected neuronal injury, or that neuronal recovery may have already occurred. Several studies do demonstrate that HO-2 deficiency (HO-2 knockout mice) associates with poor recovery from traumatic brain injury (Yoneyama-Sarnecky et al. 2010), which may be linked to increased lipid peroxidation–associated neuronal loss after such injury (Chang et al. 2003).

We also noted some specific differences in these responses among different brain regions, and we speculate that there are likely functional consequences, as has been suggested in previous studies of PLWH (Abidin et al. 2016; Chockanathan et al. 2019; Cysique et al. 2018, 2004, 2013; Kumar et al. 2009, 2011; Mohamed et al. 2018). While immunoproteasome subunit expression (LMP7, Pa28α) was higher in 7/15 brain regions, three of these regions (globus pallidus, cerebellum, and PCC) also showed significantly higher type I IFN–stimulated gene expression (*MX1*).

We speculate that our data reflect the particular vulnerability of the PCC to HIV-induced neuroinflammation, which could have consequences for neurocognitive functioning. The prevalence of deficits in executive functioning is high in PLWH, even in those not meeting the full diagnostic criteria for HIV-NCI (Correa et al. 2016). Indeed, functional neuroimaging studies have identified metabolic abnormalities in the PCC that associate with HIV infection and with progression of HIV-NCI, as well as abnormal aging and amyloid pathology in the HIV-negative population (Bladowska et al. 2013; Cysique et al. 2018; Murray et al. 2014). In a previous study of PLWH, microglial activation in PCC associated specifically with poorer executive function, suggesting clinical significance to neuroinflammation in that region even in PLWH who do not meet the strict Frascati criteria for the diagnosis of HIV-NCI (Garvey et al., 2012). Cysique et al. (2018, 2013) used magnetic resonance spectroscopy to detect neuronal injury (reduced *N*-acetyl-aspartate (NAA)) and neuroinflammation (increased myoinositol (mI)) in PCC in virally suppressed PLWH compared to HIV-negative individuals. However, we cannot attribute deficits in executive function solely to involvement of the PCC in our PLWH cohort, as such dysfunction is also affected by other brain regions, including frontal cortex and white matter, precuneus, accumbens, putamen, and globus pallidus (Correa et al. 2016; Mohamed et al. 2018).

Other subcortical brain regions have also been implicated in selective vulnerability to HIV and associated NCI. Vulnerability of the neostriatum (caudate + putamen) to HIV-associated blood-brain barrier disruption and impaired function has been observed, especially in individuals with HIV-associated dementia and HIV-encephalitis (Berger and Arendt 2000; Chaganti et al. 2019; Israel et al. 2019; Lopez et al. 1999; Melrose et al. 2008; Meltzer et al. 1998). Our study also implicates the globus pallidus, which, along with caudate and putamen, shows neuroinflammation (microglial activation) in PLWH despite a suppressive combination of antiretroviral therapy (Vera et al. 2016). Similar to our findings in the PCC, our data demonstrate neuroinflammation (*MX1*) and immunoproteasome expression (LMP7, Pa28α) in globus pallidus, extending neuropathological observations to this region within the basal ganglia (caudate + putamen + globus pallidus), and they further suggest its potential vulnerability to neuroinflammation in PLWH. Although we cannot specifically link our globus pallidus findings to NCI in our PLWH cohort, the links between basal ganglia dysfunction, neuroinflammation, and HIV-NCI are the subject of many studies (Alakkas et al. 2018; Ances et al. 2012; Aylward et al. 1993; Brew et al. 1995; Chang et al. 2004; Moore et al. 2006; Stout et al. 1998). Finally, cerebellar atrophy, degeneration, and decreased functional connectivity have also been reported in PLWH (Elsheikh et al. 2010; Tagliati et al. 1998; Wang et al. 2018).

Whether the specific neuroinflammatory changes (type I IFN–stimulated genes, endothelial activation) or immunoproteasome induction identified in this study contributes specifically to dysfunction in these brain regions in PLWH will require further investigation. We speculate that the increase of endothelial adhesion molecules might promote increased immune cell adhesion and migration through the blood-brain barrier, which could promote local neuroinflammation. The clustering of ICAM-1 and VCAM-1 at the apical surface of endothelial cells and the facilitation of leukocyte adhesion prior to diapedesis are well known (Muller 2011, 2015; Ou et al. 2008; Rahman and Fazal 2009; Rossi et al. 2011). Expression of type I IFN (IFN-α) has been linked to HIV-NCI presumably by promoting a pro-inflammatory state (Thaney and Kaul 2019). Finally, increased immunoproteasome expression in the prefrontal cortex in PLWH is associated with HIV-NCI (Gelman and Nguyen 2010; Kovacsics et al. 2017; Nguyen et al. 2010), and increased immunoproteasome expression is associated with enhanced autoimmunity and neurodegeneration in other CNS disease states (Jansen et al. 2014; Limanaqi et al. 2019). In total, our data suggest that HIV infection of the brain broadly induces neuroinflammatory responses and antioxidant responses that are more pronounced in certain regions, which may promote more global or more regionally influenced features of NCI, respectively.

We have demonstrated expression of neuroinflammatory and immunoproteasome markers that associate with expression of the antioxidant response (HO-1 expression) in multiple cortical, subcortical, and brainstem regions in PLWH without HIV-NCI. These observations extend previous findings of HIV-associated responses in the prefrontal cortex, and they suggest a ubiquitous response pattern throughout the brain to HIV infection. We speculate that the absence of synaptic protein loss in the setting of neuroinflammation may reflect a protective effect of the antioxidant response. We further speculate that more robust regional responses, such as those within the PCC, globus pallidus, and cerebellum, might disrupt normal circuitry, with effects that may be detected only with more selective clinical assessments targeting such regions. Finally, we speculate that targeting the regulation of the HO-1 antioxidant response might offer therapeutic options for neuroprotection against HIV throughout the brain.

## Electronic supplementary material

ESM 1 (DOCX 5047 kb)

## References

[CR1] Abidin AZ, D'Souza AM, Nagarajan MB, Wismuller A (2016). Investigating changes in brain network properties in HIV-associated neurocognitive disease (HAND) using mutual connectivity analysis (MCA). Proc SPIE Int Soc Opt Eng 9788 10.1117/12.2217317 PMC5697155 29170586

[CR2] Alakkas A, Ellis RJ, Watson CW, Umlauf A, Heaton RK, Letendre S, Collier A, Marra C, Clifford DB, Gelman B, Sacktor N, Morgello S, Simpson D, McCutchan JA, Kallianpur A, Gianella S, Marcotte T, Grant I, Fennema-Notestine C, CHARTER Group (2018) White matter damage, neuroinflammation, and neuronal integrity in HAND. J Neuro-Oncol 10.1007/s13365-018-0682-9 PMC6416232 30291567

[CR3] Ambegaokar SS, Kolson DL (2014). Heme oxygenase-1 dysregulation in the brain: implications for HIV-associated neurocognitive disorders. Curr HIV Res.

[CR4] Ances BM, Ortega M, Vaida F, Heaps J, Paul R (2012). Independent effects of HIV, aging, and HAART on brain volumetric measures. J Acquir Immune Defic Syndr.

[CR5] Antinori A, Arendt G, Becker JT, Brew BJ, Byrd DA, Cherner M, Clifford DB, Cinque P, Epstein LG, Goodkin K, Gisslen M, Grant I, Heaton RK, Joseph J, Marder K, Marra CM, McArthur JC, Nunn M, Price RW, Pulliam L, Robertson KR, Sacktor N, Valcour V, Wojna VE (2007). Updated research nosology for HIV-associated neurocognitive disorders. Neurology.

[CR6] Aylward EH, Henderer JD, McArthur JC, Brettschneider PD, Harris GJ, Barta PE, Pearlson GD (1993). Reduced basal ganglia volume in HIV-1-associated dementia: results from quantitative neuroimaging. Neurology.

[CR7] Bai CH, Chen JR, Chiu HC, Chou CC, Chau LY, Pan WH (2010). Shorter GT repeat polymorphism in the heme oxygenase-1 gene promoter has protective effect on ischemic stroke in dyslipidemia patients. J Biomed Sci.

[CR8] Basler M, Kirk CJ, Groettrup M (2013). The immunoproteasome in antigen processing and other immunological functions. Curr Opin Immunol.

[CR9] Berger JR, Arendt G (2000). HIV dementia: the role of the basal ganglia and dopaminergic systems. J Psychopharmacol.

[CR10] Bladowska J, Zimny A, Koltowska A, Szewczyk P, Knysz B, Gasiorowski J, Furdal M, Sasiadek MJ (2013). Evaluation of metabolic changes within the normal appearing gray and white matters in neurologically asymptomatic HIV-1-positive and HCV-positive patients: magnetic resonance spectroscopy and immunologic correlation. Eur J Radiol.

[CR11] Boerwinkle A, Ances BM (2019). Molecular imaging of neuroinflammation in HIV. J NeuroImmune Pharmacol.

[CR12] Brew BJ, Rosenblum M, Cronin K, Price RW (1995). AIDS dementia complex and HIV-1 brain infection: clinical-virological correlations. Ann Neurol.

[CR13] Cassol E, Misra V, Dutta A, Morgello S, Gabuzda D (2014). Cerebrospinal fluid metabolomics reveals altered waste clearance and accelerated aging in HIV patients with neurocognitive impairment. AIDS.

[CR14] Chaganti J, Marripudi K, Staub LP, Rae CD, Gates TM, Moffat KJ, Brew BJ (2019). Imaging correlates of the blood brain barrier disruption in HIV associated neurocognitive disorder and therapeutic implications. AIDS.

[CR15] Chang EF, Wong RJ, Vreman HJ, Igarashi T, Galo E, Sharp FR, Stevenson DK, Noble-Haeusslein LJ (2003). Heme oxygenase-2 protects against lipid peroxidation-mediated cell loss and impaired motor recovery after traumatic brain injury. J Neurosci.

[CR16] Chang L, Lee PL, Yiannoutsos CT, Ernst T, Marra CM, Richards T, Kolson D, Schifitto G, Jarvik JG, Miller EN, Lenkinski R, Gonzalez G, Navia BA (2004). A multicenter in vivo proton-MRS study of HIV-associated dementia and its relationship to age. Neuroimage.

[CR17] Chen J (2014). Heme oxygenase in neuroprotection: from mechanisms to therapeutic implications. Rev Neurosci.

[CR18] Chen J, Tu Y, Connolly EC, Ronnett GV (2005). Heme oxygenase-2 protects against glutathione depletion-induced neuronal apoptosis mediated by bilirubin and cyclic GMP. Curr Neurovasc Res.

[CR19] Chen J, Tu Y, Moon C, Nagata E, Ronnett GV (2003). Heme oxygenase-1 and heme oxygenase-2 have distinct roles in the proliferation and survival of olfactory receptor neurons mediated by cGMP and bilirubin, respectively. J Neurochem.

[CR20] Chen M, Zhou L, Ding H, Huang S, He M, Zhang X, Cheng L, Wang D, Hu FB, Wu T (2012). Short (GT) ( n ) repeats in heme oxygenase-1 gene promoter are associated with lower risk of coronary heart disease in subjects with high levels of oxidative stress. Cell Stress Chaperones.

[CR21] Chen MF, Gill AJ, Kolson DL (2014). Neuropathogenesis of HIV-associated neurocognitive disorders: roles for immune activation, HIV blipping and viral tropism. Curr Opin HIV AIDS.

[CR22] Chen YH, Lin SJ, Lin MW, Tsai HL, Kuo SS, Chen JW, Charng MJ, Wu TC, Chen LC, Ding YA, Pan WH, Jou YS, Chau LY (2002). Microsatellite polymorphism in promoter of heme oxygenase-1 gene is associated with susceptibility to coronary artery disease in type 2 diabetic patients. Hum Genet.

[CR23] Chockanathan U, AM DS, Abidin AZ, Schifitto G, Wismüller A (2019). Automated diagnosis of HIV-associated neurocognitive disorders using large-scale Granger causality analysis of resting-state functional MRI. Comput Biol Med.

[CR24] Correa DG, Zimmermann N, Netto TM, Tukamoto G, Ventura N, de Castro Bellini Leite S, Cabral RF, Fonseca RP, Bahia PR, Gasparetto EL (2016). Regional cerebral gray matter volume in HIV-positive patients with executive function deficits. J Neuroimaging.

[CR25] Cross SA, Cook DR, Chi AW, Vance PJ, Kolson LL, Wong BJ, Jordan-Sciutto KL, Kolson DL (2011). Dimethyl fumarate, an immune modulator and inducer of the antioxidant response, suppresses HIV replication and macrophage-mediated neurotoxicity: a novel candidate for HIV neuroprotection. J Immunol.

[CR26] Cysique LA, Juge L, Gates T, Tobia M, Moffat K, Brew BJ, Rae C (2018). Covertly active and progressing neurochemical abnormalities in suppressed HIV infection. Neurol Neuroimmunol Neuroinflamm.

[CR27] Cysique LA, Maruff P, Brew BJ (2004). Prevalence and pattern of neuropsychological impairment in human immunodeficiency virus-infected/acquired immunodeficiency syndrome (HIV/AIDS) patients across pre- and post-highly active antiretroviral therapy eras: a combined study of two cohorts. J Neuro-Oncol.

[CR28] Cysique LA, Moffat K, Moore DM, Lane TA, Davies NW, Carr A, Brew BJ, Rae C (2013). HIV, vascular and aging injuries in the brain of clinically stable HIV-infected adults: a (1)H MRS study. PLoS One.

[CR29] Dore S, Goto S, Sampei K, Blackshaw S, Hester LD, Ingi T, Sawa A, Traystman RJ, Koehler RC, Snyder SH (2000). Heme oxygenase-2 acts to prevent neuronal death in brain cultures and following transient cerebral ischemia. Neuroscience.

[CR30] Dore S, Sampei K, Goto S, Alkayed NJ, Guastella D, Blackshaw S, Gallagher M, Traystman RJ, Hurn PD, Koehler RC, Snyder SH (1999). Heme oxygenase-2 is neuroprotective in cerebral ischemia. Mol Med.

[CR31] Elsheikh BH, Maher WE, Kissel JT (2010). Cerebellar atrophy associated with human immunodeficiency virus infection. Arch Neurol.

[CR32] Espey MG, Basile AS, Heaton RK, Ellis RJ (2002). Increased glutamate in CSF and plasma of patients with HIV dementia. Neurology.

[CR33] Espey MG, Ellis RJ, Heaton RK, Basile AS (1999). Relevance of glutamate levels in the CSF of patients with HIV-1-associated dementia complex. Neurology.

[CR34] Ferrarese C, Aliprandi A, Tremolizzo L, Stanzani L, De Micheli A, Dolara A, Frattola L (2001). Increased glutamate in CSF and plasma of patients with HIV dementia. Neurology.

[CR35] Fruh K, Gossen M, Wang K, Bujard H, Peterson PA, Yang Y (1994). Displacement of housekeeping proteasome subunits by MHC-encoded LMPs: a newly discovered mechanism for modulating the multicatalytic proteinase complex. EMBO J.

[CR36] Garvey L, Pavese, N.; Politis, M.; Ramlackhansingh, A.; Taylor-Robinson, SD.; Brooks, D.; Winston, A. (2012). Microglial cell activation is visualized with 11C-[R]-PK11195-PET scans in neuro-asymptomatic HIV infected subjects on effective antiretroviral therapy. In: Conference on retroviruses and opportunistic infections. Seattle, WA

[CR37] Garza R, Gill AJ, Bastien BL, Garcia-Mesa Y, Gruenewald AL, Gelman BB, Tsima B, Gross R, Letendre SL, Kolson DL (2020). Heme oxygenase-1 promoter (GT) n polymorphism associates with HIV neurocognitive impairment. Neurol Neuroimmunol Neuroinflamm.

[CR38] Gelman BB (2015). Neuropathology of HAND with suppressive antiretroviral therapy: encephalitis and neurodegeneration reconsidered. Curr HIV/AIDS Rep.

[CR39] Gelman BB, Chen T, Lisinicchia JG, Soukup VM, Carmical JR, Starkey JM, Masliah E, Commins DL, Brandt D, Grant I, Singer EJ, Levine AJ, Miller J, Winkler JM, Fox HS, Luxon BA, Morgello S, National Neuro ATC (2012). The National NeuroAIDS Tissue Consortium brain gene array: two types of HIV-associated neurocognitive impairment. PLoS One.

[CR40] Gelman BB, Endsley J, Kolson D (2018). When do models of NeuroAIDS faithfully imitate “the real thing”?. J Neuro-Oncol.

[CR41] Gelman BB, Lisinicchia JG, Chen T, Johnson KM, Jennings K, Freeman DH, Soukup VM (2012). Prefrontal dopaminergic and enkephalinergic synaptic accommodation in HIV-associated neurocognitive disorders and encephalitis. J NeuroImmune Pharmacol.

[CR42] Gelman BB, Nguyen TP (2010). Synaptic proteins linked to HIV-1 infection and immunoproteasome induction: proteomic analysis of human synaptosomes. J NeuroImmune Pharmacol.

[CR43] Gill AJ, Garza R, Ambegaokar SS, Gelman BB, Kolson DL (2018). Heme oxygenase-1 promoter region (GT)n polymorphism associates with increased neuroimmune activation and risk for encephalitis in HIV infection. J Neuroinflammation.

[CR44] Gill AJ, Kovacsics CE, Cross SA, Vance PJ, Kolson LL, Jordan-Sciutto KL, Gelman BB, Kolson DL (2014). Heme oxygenase-1 deficiency accompanies neuropathogenesis of HIV-associated neurocognitive disorders. J Clin Invest.

[CR45] Gill AJ, Kovacsics CE, Vance PJ, Collman RG, Kolson DL (2015). Induction of heme oxygenase-1 deficiency and associated glutamate-mediated neurotoxicity is a highly conserved HIV phenotype of chronic macrophage infection that is resistant to antiretroviral therapy. J Virol.

[CR46] Han F, Takeda K, Yokoyama S, Ueda H, Shinozawa Y, Furuyama K, Shibahara S (2005). Dynamic changes in expression of heme oxygenases in mouse heart and liver during hypoxia. Biochem Biophys Res Commun.

[CR47] He JZ, Ho JJ, Gingerich S, Courtman DW, Marsden PA, Ward ME (2010). Enhanced translation of heme oxygenase-2 preserves human endothelial cell viability during hypoxia. J Biol Chem.

[CR48] Hill J, Rom S, Ramirez SH, Persidsky Y (2014). Emerging roles of pericytes in the regulation of the neurovascular unit in health and disease. J NeuroImmune Pharmacol.

[CR49] Hirai H, Kubo H, Yamaya M, Nakayama K, Numasaki M, Kobayashi S, Suzuki S, Shibahara S, Sasaki H (2003). Microsatellite polymorphism in heme oxygenase-1 gene promoter is associated with susceptibility to oxidant-induced apoptosis in lymphoblastoid cell lines. Blood.

[CR50] Hong S, Banks WA (2015). Role of the immune system in HIV-associated neuroinflammation and neurocognitive implications. Brain Behav Immun.

[CR51] Israel SM, Hassanzadeh-Behbahani S, Turkeltaub PE, Moore DJ, Ellis RJ, Jiang X (2019). Different roles of frontal versus striatal atrophy in HIV-associated neurocognitive disorders. Hum Brain Mapp.

[CR52] Jansen AH, Reits EA, Hol EM (2014). The ubiquitin proteasome system in glia and its role in neurodegenerative diseases. Front Mol Neurosci.

[CR53] Jimenez-Guardeno JM, Apolonia L, Betancor G, Malim MH (2019). Immunoproteasome activation enables human TRIM5alpha restriction of HIV-1. Nat Microbiol.

[CR54] Kaartokallio T, Utge S, Klemetti MM, Paananen J, Pulkki K, Romppanen J, Tikkanen I, Heinonen S, Kajantie E, Kere J, Kivinen K, Pouta A, Lakkisto P, Laivuori H (2018). Fetal microsatellite in the heme oxygenase 1 promoter is associated with severe and early-onset preeclampsia. Hypertension.

[CR55] Kovacsics CE, Gill AJ, Ambegaokar SS, Gelman BB, Kolson DL (2017). Degradation of heme oxygenase-1 by the immunoproteasome in astrocytes: a potential interferon-gamma-dependent mechanism contributing to HIV neuropathogenesis. Glia.

[CR56] Kumar AM, Fernandez JB, Singer EJ, Commins D, Waldrop-Valverde D, Ownby RL, Kumar M (2009). Human immunodeficiency virus type 1 in the central nervous system leads to decreased dopamine in different regions of postmortem human brains. J Neuro-Oncol.

[CR57] Kumar AM, Ownby RL, Waldrop-Valverde D, Fernandez B, Kumar M (2011). Human immunodeficiency virus infection in the CNS and decreased dopamine availability: relationship with neuropsychological performance. J Neuro-Oncol.

[CR58] Limanaqi F, Biagioni F, Gaglione A, Busceti CL, Fornai F (2019). A sentinel in the crosstalk between the nervous and immune system: the (immuno)-proteasome. Front Immunol.

[CR59] Lopez OL, Smith G, Meltzer CC, Becker JT (1999). Dopamine systems in human immunodeficiency virus-associated dementia. Neuropsychiatry Neuropsychol Behav Neurol.

[CR60] Melrose RJ, Tinaz S, Castelo JM, Courtney MG, Stern CE (2008). Compromised fronto-striatal functioning in HIV: an fMRI investigation of semantic event sequencing. Behav Brain Res.

[CR61] Meltzer CC, Wells SW, Becher MW, Flanigan KM, Oyler GA, Lee RR (1998). AIDS-related MR hyperintensity of the basal ganglia. AJNR Am J Neuroradiol.

[CR62] Mohamed M, Barker PB, Skolasky RL, Sacktor N (2018). 7T brain MRS in HIV infection: correlation with cognitive impairment and performance on neuropsychological tests. AJNR Am J Neuroradiol.

[CR63] Moore DJ, Masliah E, Rippeth JD, Gonzalez R, Carey CL, Cherner M, Ellis RJ, Achim CL, Marcotte TD, Heaton RK, Grant I, HNRC Group (2006). Cortical and subcortical neurodegeneration is associated with HIV neurocognitive impairment. AIDS.

[CR64] Muller WA (2011). Mechanisms of leukocyte transendothelial migration. Annu Rev Pathol.

[CR65] Muller WA (2015). The regulation of transendothelial migration: new knowledge and new questions. Cardiovasc Res.

[CR66] Murray ME, Przybelski SA, Lesnick TG, Liesinger AM, Spychalla A, Zhang B, Gunter JL, Parisi JE, Boeve BF, Knopman DS, Petersen RC, Jack CR, Dickson DW, Kantarci K (2014). Early Alzheimer’s disease neuropathology detected by proton MR spectroscopy. J Neurosci.

[CR67] Nandi D, Woodward E, Ginsburg DB, Monaco JJ (1997). Intermediates in the formation of mouse 20S proteasomes: implications for the assembly of precursor beta subunits. EMBO J.

[CR68] Nguyen TP, Soukup VM, Gelman BB (2010). Persistent hijacking of brain proteasomes in HIV-associated dementia. Am J Pathol.

[CR69] Ou R, Zhang M, Huang L, Flavell RA, Koni PA, Moskophidis D (2008). Regulation of immune response and inflammatory reactions against viral infection by VCAM-1. J Virol.

[CR70] Parfenova H, Basuroy S, Bhattacharya S, Tcheranova D, Qu Y, Regan RF, Leffler CW (2006). Glutamate induces oxidative stress and apoptosis in cerebral vascular endothelial cells: contributions of HO-1 and HO-2 to cytoprotection. Am J Physiol Cell Physiol.

[CR71] Peluso MJ, Meyerhoff DJ, Price RW, Peterson J, Lee E, Young AC, Walter R, Fuchs D, Brew BJ, Cinque P, Robertson K, Hagberg L, Zetterberg H, Gisslen M, Spudich S (2013). Cerebrospinal fluid and neuroimaging biomarker abnormalities suggest early neurological injury in a subset of individuals during primary HIV infection. J Infect Dis.

[CR72] Pickering AM, Koop AL, Teoh CY, Ermak G, Grune T, Davies KJ (2010). The immunoproteasome, the 20S proteasome and the PA28alphabeta proteasome regulator are oxidative-stress-adaptive proteolytic complexes. Biochem J.

[CR73] Rahman A, Fazal F (2009). Hug tightly and say goodbye: role of endothelial ICAM-1 in leukocyte transmigration. Antioxid Redox Signal.

[CR74] Rossi B, Angiari S, Zenaro E, Budui SL, Constantin G (2011). Vascular inflammation in central nervous system diseases: adhesion receptors controlling leukocyte-endothelial interactions. J Leukoc Biol.

[CR75] Rueda B, Oliver J, Robledo G, Lopez-Nevot MA, Balsa A, Pascual-Salcedo D, Gonzalez-Gay MA, Gonzalez-Escribano MF, Martin J (2007). HO-1 promoter polymorphism associated with rheumatoid arthritis. Arthritis Rheum.

[CR76] Sailasuta N, Ross W, Ananworanich J, Chalermchai T, DeGruttola V, Lerdlum S, Pothisri M, Busovaca E, Ratto-Kim S, Jagodzinski L, Spudich S, Michael N, Kim JH, Valcour V, RV254/SEARCH 010 protocol teams (2012). Change in brain magnetic resonance spectroscopy after treatment during acute HIV infection. PLoS One.

[CR77] Saing T, Lagman M, Castrillon J, Gutierrez E, Guilford FT, Venketaraman V (2016). Analysis of glutathione levels in the brain tissue samples from HIV-1-positive individuals and subject with Alzheimer’s disease and its implication in the pathophysiology of the disease process. BBA Clin.

[CR78] Saylor D, Dickens AM, Sacktor N, Haughey N, Slusher B, Pletnikov M, Mankowski JL, Brown A, Volsky DJ, McArthur JC (2016). HIV-associated neurocognitive disorder - pathogenesis and prospects for treatment. Nat Rev Neurol.

[CR79] Schipper HM (2004). Heme oxygenase expression in human central nervous system disorders. Free Radic Biol Med.

[CR80] Schipper HM, Song W (2015). A heme oxygenase-1 transducer model of degenerative and developmental brain disorders. Int J Mol Sci.

[CR81] Seu L, Burt TD, Witte JS, Martin JN, Deeks SG, McCune JM (2012). Variations in the heme oxygenase-1 microsatellite polymorphism are associated with plasma CD14 and viral load in HIV-infected African-Americans. Genes Immun.

[CR82] Shevchenko A, Wilm M, Vorm O, Mann M (1996). Mass spectrometric sequencing of proteins silver-stained polyacrylamide gels. Anal Chem.

[CR83] Spudich SS (2016). Immune activation in the central nervous system throughout the course of HIV infection. Curr Opin HIV AIDS.

[CR84] Stout JC, Ellis RJ, Jernigan TL, Archibald SL, Abramson I, Wolfson T, McCutchan JA, Wallace MR, Atkinson JH, Grant I (1998). Progressive cerebral volume loss in human immunodeficiency virus infection: a longitudinal volumetric magnetic resonance imaging study. HIV Neurobehavioral Research Center Group. Arch Neurol.

[CR85] Tagliati M, Simpson D, Morgello S, Clifford D, Schwartz RL, Berger JR (1998). Cerebellar degeneration associated with human immunodeficiency virus infection. Neurology.

[CR86] Takahashi K, Hara E, Suzuki H, Sasano H, Shibahara S (1996). Expression of heme oxygenase isozyme mRNAs in the human brain and induction of heme oxygenase-1 by nitric oxide donors. J Neurochem.

[CR87] Tavazzi E, Morrison D, Sullivan P, Morgello S, Fischer T (2014). Brain inflammation is a common feature of HIV-infected patients without HIV encephalitis or productive brain infection. Curr HIV Res.

[CR88] Thaney VE, Kaul M (2019). Type I interferons in NeuroHIV. Viral Immunol.

[CR89] Tremolizzo L, Aliprandi A, Longoni M, Stanzani L, Ferrarese C (2002). Glutamate may be the soluble cerebrospinal fluid factor that induces calcium dysregulation in cultured astrocytes in HIV dementia. Aids.

[CR90] Vázquez-Armenta G, González-Leal N, Vázquez-de la Torre MJ, Muñoz-Valle JF, Ramos-Márquez ME, Hernández-Cañaveral I, Plascencia-Hernández A, Siller-López F (2013). Short (GT)n microsatellite repeats in the heme oxygenase-1 gene promoter are associated with antioxidant and anti-inflammatory status in Mexican pediatric patients with sepsis. Tohoku J Exp Med.

[CR91] Vera JH, Guo Q, Cole JH, Boasso A, Greathead L, Kelleher P, Rabiner EA, Kalk N, Bishop C, Gunn RN, Matthews PM, Winston A (2016). Neuroinflammation in treated HIV-positive individuals: a TSPO PET study. Neurology.

[CR92] Vilander LM, Vaara ST, Donner KM, Lakkisto P, Kaunisto MA, Pettila V, FINNAKI Study Group (2019). Heme oxygenase-1 repeat polymorphism in septic acute kidney injury. PLoS One.

[CR93] Vukomanovic D, McLaughlin BE, Rahman MN, Szarek WA, Brien JF, Jia Z, Nakatsu K (2011). Selective activation of heme oxygenase-2 by menadione. Can J Physiol Pharmacol.

[CR94] Wang H, Li R, Zhou Y, Wang Y, Cui J, Nguchu BA, Qiu B, Wang X, Li H (2018). Altered cerebro-cerebellum resting-state functional connectivity in HIV-infected male patients. J Neuro-Oncol.

[CR95] Wang J, Zhuang H, Dore S (2006). Heme oxygenase 2 is neuroprotective against intracerebral hemorrhage. Neurobiol Dis.

[CR96] Wu B, Wu Y, Tang W (2019). Heme catabolic pathway in inflammation and immune disorders. Front Pharmacol.

[CR97] Yoneyama-Sarnecky T, Olivas AD, Azari S, Ferriero DM, Manvelyan HM, Noble-Haeusslein LJ (2010). Heme oxygenase-2 modulates early pathogenesis after traumatic injury to the immature brain. Dev Neurosci.

[CR98] Zhang Y, Furuyama K, Kaneko K, Ding Y, Ogawa K, Yoshizawa M, Kawamura M, Takeda K, Yoshida T, Shibahara S (2006). Hypoxia reduces the expression of heme oxygenase-2 in various types of human cell lines. A possible strategy for the maintenance of intracellular heme level. FEBS J.

