# Supplementary material for: Neuroinflammation associates with antioxidant heme oxygenase-1 response throughout the brain in persons living with HIV
Source: J Neurovirol. 2020 Sep 10;26(6):846–62. doi: 10.1007/s13365-020-00902-8 (PMC7716923; doi:10.1007/s13365-020-00902-8)
Supplement: Supplementary file 1 — (DOCX 5047 kb) [file 13365_2020_902_MOESM1_ESM.docx]

**Supplementary Figures**

**Supplementary Fig. 1** (**a**) Human brain tissue lysates were probed for β-tubulin and HO-1 (Enzo ADI-SPA-895) by Western blot. For a control comparator, we used differentiated human monocyte-derived macrophages (MDMs), which express high levels of HO-1 after siRNA knock-down of the Nrf2 repressor protein BACH-1 (Gill *et al*, 2014; Sun *et al*, 2002). MDMs were treated with siRNAs targeting HO-1 or BACH1 for 6 days. In human brain lysates, we observed two bands at ~32kDa (white and black arrows), of which the higher molecular weight band (white arrow) was consistently more intense. In human MDMs, we observed the same two bands, but only the lower molecular weight band (black arrow) was enhanced as expected by anti-BACH1 siRNA transfection. (**b**) Human brain lysate was resolved by SDS-PAGE in two identically loaded lanes. One half of SDS-PAGE gel was stained with Coomassie blue (left) and the other was transferred to PVDF membrane and probed for HO-1 with ADI-SPA-895 (right). Two bands were dissected from the stained gel (left) based on location of bands in 32kDa range on the membrane (white and black arrows), trypsin-digested, and subjected to mass spectrometric analysis. Sequence-to-spectrum peptide assignments generated by SEQUEST in Proteome Discoverer were loaded into Scaffold to validate MS/MS peptide and protein identification. Four unique peptides with Xcorr scores >2.0 and delta Cn >0.1 representing 21% coverage of the HO1 sequence were identified in the two gel bands. Each MS/MS spectrum was also manually evaluated and contained contiguous series of y-series fragment ions. (**c**) Human and wild-type C57Bl/6 (WT), HO-1 knockout (HO-1 KO), and human HO-1 overexpressing (HO-1 hBAC) mouse brain and spleen lysates (courtesy of Dr. Anupam Agarwal, UAB) were probed for β-tubulin and HO-1 using Enzo ADI-SPA-895. Two bands were detected at ~32kDa in human brain tissue lysates (white and black arrows), and a similar banding pattern in transgenic mouse brain tissue lysates. The higher molecular weight band (white arrow) was detected in HO-1 knockout mouse brain tissue lysates, indicating a protein other than HO-1 at 32kDa. No such band was observed in spleen lysates from mouse or human, or in the HO-1 knockout mouse lysates. These results suggest that ADI-SPA-895 detects HO-1 protein in both bands, as well as another protein in the higher molecular weight band. (**d**) Human and wild-type C57Bl/6 (WT), HO-1 knockout (HO-1 KO), and human HO-1 overexpressing (HO-1 hBAC) mouse brain and spleen lysates were probed for β-tubulin and HO-1 using Enzo ADI-SPA-894. A single band was detected at 32kDa in mouse brain tissue lysates (black arrow), which was not present in HO-1 knockout mouse brain or spleen. We confirmed HO-1 content in this band, and therefore used this antibody in all subsequent experiments.

**Supplementary Fig. 2** (**a**) HO-1 protein and (**b**) RNA expression were measured by Western blot and RT-qPCR, respectively. PLWH were stratified by promoter genotype (orange circles: short/short [S/S]; blue squares: long/long [L/L]). (**a, c**) Effect of HO-1 (GT)n promoter genotype on HO-1 expression in individual brain regions was measured by Student’s unpaired t-test. (**b, d**) Effect of HO-1 (GT)_n_ promoter genotype on whole brain HO-1 expression was measured by Student’s paired t-test. *p<0.05, ** p<0.01, ***p<0.001, ****p<0.0001

**
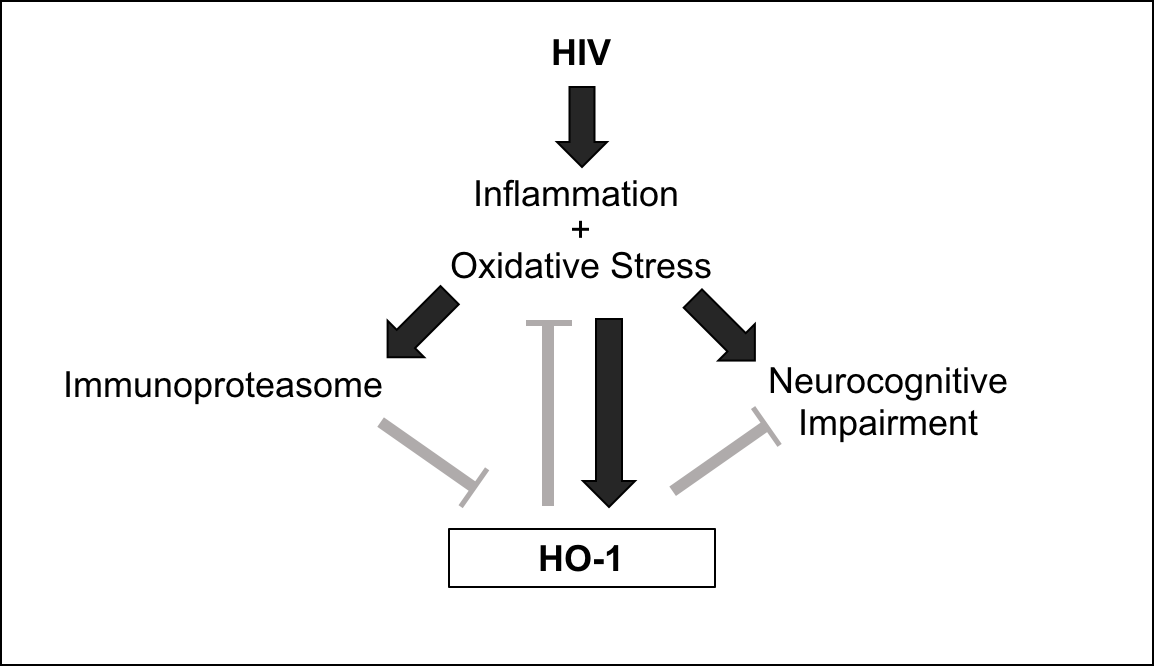
**

**Supplementary Fig. 3** Schematic depicting proposed relationships between inflammation and oxidative stress, HO-1, immunoproteasome, and NCI in PLWH. HIV infection increases inflammation and oxidative stress, which associates with HIV NCI. Inflammation and oxidative stress also stimulate immunoproteasome and HO-1 expression. HO-1 induction limits inflammation, oxidative stress, and may protect against HIV NCI. Immunoproteasome induction drives down HO-1, decrease of which associates with HIV NCI.

**Supplementary Tables**

**Supplementary Table 1** CNS regions analyzed and abbreviations

| **Regions Analyzed** | **Abbreviation** |
| --- | --- |
| Frontal Cortex | FC |
| Temporal Cortex | TC |
| Occipital Cortex | OC |
| Motor Cortex | MC |
| Sensory Cortex | SC |
| Anterior Cingulate Cortex | ACC |
| Posterior Cingulate Cortex | PCC |
| Amygdala | AM |
| Caudate Nucleus/Putamen | CN |
| Globus Pallidus | GP |
| Pons | PN |
| Midbrain | MB |
| Medulla | MED |
| Cerebellum | CB |
| Frontal White Matter | FWM |
| Spinal Cord | SPC |

**Supplementary Table 2** Primary antibodies used for Western blot

| **Primary Antibodies** | |  | |  | |  | | |
| --- | --- | --- | --- | --- | --- | --- | --- | --- |
| **Antibody** | **Type** | | **Source** | | **Catalog #** | | **Dilution** |  |
| β-tubulin | Rabbit mAb | | Cell Signaling Technology | | 2128L | | 1:3000 |  |
| Proteasome subunit LMP7 | Mouse mAb | | Enzo Life Sciences | | BML-PW8845 | | 1:500 |  |
| Proteasome subunit β5 | Rabbit pAb | | Invitrogen | | PA1-977 | | 1:1000 |  |
| GAPDH | Mouse mAb | | Calbiochem | | CB1001 | | 1:20000 |  |
| VCAM-1 | Rabbit mAb | | Abcam | | ab134047 | | 1:2000 |  |
| PECAM-1 | Rabbit pAb | | Abcam | | ab28364 | | 1:500 |  |
| ICAM-1 | Rabbit mAb | | Abcam | | ab109361 | | 1:1000 |  |
| PSD95 | Mouse mAb | | EMD Millipore | | MAB1596 | | 1:1000 |  |
| Synaptophysin | Mouse mAb | | Abcam | | Ab8049 | | 1:1000 |  |
| HO-2 | Rabbit pAb | | Enzo Life Sciences | | SPA-897 | | 1:1000 |  |
| HO-1 | Rabbit pAb | | Enzo Life Sciences | | SPA-895 | | 1:500 |  |
| HO-1 | Rabbit pAb | | Enzo Life Sciences | | SPA-894 | | 1:500 |  |
| Proteasome subunit Pa28α | Rabbit pAb | | Enzo Life Sciences | | BML-PW8185 | | 1:1000 |  |

Abbreviations: monoclonal antibody (mAb); polyclonal antibody (pAb).

**Supplementary Table 3** Secondary antibodies used for Western blot

| **Secondary Antibodies** | |  |  |  |
| --- | --- | --- | --- | --- |
| **Antibody** | **Type** | **Source** | **Catalog #** | **Dilution** |
| IRDye 680RD | Goat anti-mouse IgG | LI-COR Biosciences | 926-68070 | 1:20,000 |
| IRDye 680RD | Goat anti-rabbit IgG | LI-COR Biosciences | 926-68071 | 1:20,000 |
| IRDye 800CW | Goat anti-mouse IgG | LI-COR Biosciences | 926-32210 | 1:15,000 |
| IRDye 800CW | Goat anti-rabbit IgG | LI-COR Biosciences | 926-32211 | 1:15,000 |

**Supplementary Table 4** Associations between brain HO-1 and immunoproteasome subunit LMP7 in PLWH and HIV-negative individuals

|  | **HO-1 RNA vs. LMP7 protein** | | | | **HO-1 protein vs. LMP7 protein** | | | |
| --- | --- | --- | --- | --- | --- | --- | --- | --- |
|  | **HIV-negative** | | **PLWH** | | **HIV-negative** | | **PLWH** | |
| **Region** | **Pearson r** | **p-value** | **Pearson r** | **p-value** | **Pearson r** | **p-value** | **Pearson r** | **p-value** |
| FC | -0.114 | 0.830 | 0.288 | 0.452 | 0.360 | 0.428 | 0.676 | ***0.046*** |
| TC | -0.229 | 0.711 | 0.202 | 0.602 | 0.219 | 0.637 | 0.474 | 0.198 |
| OC | 0.686 | 0.089 | 0.295 | 0.441 | 0.374 | 0.408 | 0.213 | 0.582 |
| MC | 0.754 | 0.050 | 0.003 | 0.994 | 0.190 | 0.683 | -0.094 | 0.810 |
| SC | 0.011 | 0.984 | 0.105 | 0.789 | 0.111 | 0.812 | -0.093 | 0.812 |
| ACC | 0.688 | 0.088 | 0.378 | 0.317 | 0.129 | 0.783 | 0.202 | 0.602 |
| PCC | 0.109 | 0.8163 | 0.065 | 0.8681 | 0.400 | 0.3745 | 0.289 | 0.4506 |
| AM | 0.559 | 0.192 | 0.009 | 0.982 | 0.827 | ***0.022*** | -0.134 | 0.732 |
| CN | 0.180 | 0.699 | 0.023 | 0.953 | 0.065 | 0.890 | 0.068 | 0.863 |
| GP | 0.056 | 0.905 | 0.195 | 0.615 | -0.665 | 0.104 | 0.372 | 0.324 |
| PN | -0.265 | 0.566 | 0.552 | 0.124 | 0.092 | 0.844 | 0.347 | 0.360 |
| MB | -0.173 | 0.711 | 0.014 | 0.972 | 0.623 | 0.135 | 0.151 | 0.698 |
| MED | 0.095 | 0.839 | 0.449 | 0.264 | 0.635 | 0.125 | 0.339 | 0.412 |
| CB | 0.151 | 0.775 | -0.446 | 0.229 | 0.169 | 0.718 | 0.232 | 0.549 |
| FWM | -0.224 | 0.629 | -0.239 | 0.535 | 0.568 | 0.183 | -0.247 | 0.521 |
| SPC | 0.147 | 0.814 | 0.885 | ***0.008*** | 0.558 | 0.193 | 0.627 | 0.096 |

Correlations between HO-1 RNA or protein and LMP7 in individual regions were analyzed by Pearson’s correlation. Bold and italicized text: p<0.05

**Supplementary Table 5** Associations between brain HO-1 and type I IFN-stimulated genes in HIV-negative individuals

|  | ***ISG15* RNA vs. HO-1 RNA** | | ***ISG15* RNA vs. HO-1 protein** | | ***MX1* RNA vs. HO-1 RNA** | | ***MX1* RNA vs. HO-1 protein** | |
| --- | --- | --- | --- | --- | --- | --- | --- | --- |
| **Region** | **Pearson r** | **p-value** | **Pearson r** | **p-value** | **Pearson r** | **p-value** | **Pearson r** | **p-value** |
| FC | -0.100 | 0.850 | -0.276 | 0.597 | -0.446 | 0.376 | -0.680 | 0.138 |
| TC | -0.097 | 0.876 | -0.602 | 0.282 | -0.189 | 0.761 | -0.920 | ***0.027*** |
| OC | 0.068 | 0.884 | -0.412 | 0.358 | -0.185 | 0.691 | -0.567 | 0.184 |
| MC | 0.165 | 0.724 | -0.110 | 0.815 | -0.100 | 0.831 | -0.370 | 0.414 |
| SC | 0.099 | 0.852 | -0.293 | 0.573 | 0.214 | 0.684 | -0.478 | 0.337 |
| ACC | 0.077 | 0.871 | -0.584 | 0.169 | -0.292 | 0.526 | -0.617 | 0.140 |
| PCC | -0.163 | 0.727 | -0.061 | 0.896 | -0.264 | 0.567 | -0.229 | 0.6218 |
| AM | 0.102 | 0.828 | -0.130 | 0.781 | 0.082 | 0.862 | -0.118 | 0.801 |
| CN | -0.630 | 0.130 | -0.025 | 0.958 | -0.279 | 0.544 | 0.390 | 0.387 |
| GP | -0.569 | 0.182 | -0.374 | 0.408 | -0.773 | ***0.042*** | -0.110 | 0.814 |
| PN | -0.385 | 0.394 | 0.110 | 0.815 | -0.383 | 0.396 | -0.222 | 0.632 |
| MB | -0.427 | 0.339 | -0.065 | 0.889 | -0.550 | 0.201 | 0.305 | 0.507 |
| MED | 0.558 | 0.193 | 0.210 | 0.651 | 0.654 | 0.111 | 0.350 | 0.441 |
| CB | -0.484 | 0.331 | -0.029 | 0.956 | -0.732 | 0.098 | -0.729 | 0.101 |
| FWM | -0.079 | 0.867 | -0.346 | 0.447 | -0.553 | 0.198 | -0.632 | 0.128 |
| SPC | -0.248 | 0.687 | -0.686 | 0.201 | -0.241 | 0.696 | -0.708 | 0.181 |

Correlations between HO-1 RNA or protein and *ISG15* or *MX1* RNA in individual regions were analyzed by Pearson’s correlation. Bold and italicized text: p<0.05

**Supplementary Table 6** Associations between brain HO-1 and endothelial adhesion molecules in HIV-negative individuals

|  | **ICAM-1 protein vs. HO-1 RNA** | | **ICAM-1 protein vs. HO-1 protein** | | **VCAM-1 protein vs. HO-1 RNA** | | **VCAM-1 protein vs. HO-1 protein** | | **PECAM-1 protein vs. HO-1 RNA** | | **PECAM-1 protein vs. HO-1 protein** | |
| --- | --- | --- | --- | --- | --- | --- | --- | --- | --- | --- | --- | --- |
| **Region** | **Pearson r** | **p-value** | **Pearson r** | **p-value** | **Pearson r** | **p-value** | **Pearson r** | **p-value** | **Pearson r** | **p-value** | **Pearson r** | **p-value** |
| FC | -0.650 | 0.163 | 0.117 | 0.803 | -0.166 | 0.753 | 0.420 | 0.349 | -0.511 | 0.301 | 0.609 | 0.147 |
| TC | 0.642 | 0.243 | 0.155 | 0.741 | -0.249 | 0.687 | 0.464 | 0.294 | 0.284 | 0.643 | 0.511 | 0.242 |
| OC | 0.625 | 0.133 | 0.443 | 0.319 | -0.308 | 0.501 | 0.265 | 0.566 | 0.297 | 0.518 | 0.292 | 0.526 |
| MC | 0.293 | 0.523 | 0.013 | 0.978 | -0.597 | 0.157 | -0.018 | 0.970 | 0.551 | 0.200 | 0.172 | 0.713 |
| SC | -0.776 | 0.070 | -0.317 | 0.489 | -0.774 | 0.071 | 0.294 | 0.522 | -0.451 | 0.370 | -0.230 | 0.619 |
| ACC | 0.200 | 0.668 | 0.629 | 0.130 | -0.477 | 0.279 | 0.674 | 0.097 | -0.129 | 0.784 | 0.363 | 0.424 |
| PCC | -0.283 | 0.538 | -0.221 | 0.634 | -0.183 | 0.695 | -0.035 | 0.9412 | 0.401 | 0.373 | 0.752 | 0.051 |
| AM | -0.083 | 0.860 | 0.255 | 0.581 | -0.311 | 0.498 | 0.439 | 0.238 | -0.240 | 0.604 | 0.184 | 0.694 |
| CN | 0.418 | 0.351 | 0.543 | 0.208 | 0.312 | 0.496 | 0.443 | 0.319 | 0.161 | 0.730 | 0.017 | 0.972 |
| GP | -0.391 | 0.386 | 0.024 | 0.960 | -0.350 | 0.441 | -0.326 | 0.476 | 0.105 | 0.824 | -0.084 | 0.857 |
| PN | 0.230 | 0.621 | 0.961 | ***0.001*** | 0.145 | 0.756 | -0.466 | 0.292 | -0.100 | 0.832 | 0.784 | ***0.037*** |
| MB | -0.030 | 0.948 | 0.104 | 0.824 | -0.609 | 0.147 | 0.379 | 0.402 | -0.096 | 0.838 | 0.129 | 0.783 |
| MED | -0.079 | 0.866 | 0.426 | 0.341 | -0.062 | 0.896 | 0.199 | 0.669 | -0.608 | 0.148 | 0.190 | 0.684 |
| CB | 0.100 | 0.851 | 0.004 | 0.993 | -0.780 | 0.067 | 0.400 | 0.374 | -0.418 | 0.409 | 0.468 | 0.290 |
| FWM | -0.408 | 0.364 | 0.300 | 0.513 | -0.667 | 0.102 | 0.389 | 0.388 | -0.586 | 0.167 | 0.565 | 0.186 |
| SPC | 0.913 | ***0.030*** | 0.238 | 0.607 | -0.488 | 0.404 | 0.000 | 0.999 | 0.162 | 0.795 | 0.368 | 0.417 |

Correlations between HO-1 RNA or protein and ICAM-1, VCAM-1, or PECAM-1 protein in individual regions were analyzed by Pearson’s correlation. Bold and italicized text: p<0.05

**Supplementary Table 7** Associations between brain HO-1 and synaptic markers in PLWH

|  | **PSD95 protein vs. HO-1 RNA** | | **PSD95 protein vs. HO-1 protein** | | **Synaptophysin protein vs. HO-1 RNA** | | **Synaptophysin protein vs. HO-1 protein** | |
| --- | --- | --- | --- | --- | --- | --- | --- | --- |
| **Region** | **Pearson r** | **p-value** | **Pearson r** | **p-value** | **Pearson r** | **p-value** | **Pearson r** | **p-value** |
| FC | 0.168 | 0.666 | -0.063 | 0.872 | -0.297 | 0.437 | 0.309 | 0.419 |
| TC | 0.396 | 0.291 | 0.171 | 0.660 | -0.073 | 0.853 | 0.097 | 0.804 |
| OC | 0.647 | 0.060 | 0.666 | 0.050 | -0.258 | 0.504 | -0.526 | 0.145 |
| MC | 0.125 | 0.749 | 0.123 | 0.753 | -0.184 | 0.635 | -0.103 | 0.792 |
| SC | 0.433 | 0.244 | -0.011 | 0.979 | -0.293 | 0.445 | -0.447 | 0.228 |
| ACC | 0.508 | 0.162 | 0.597 | 0.089 | -0.222 | 0.566 | -0.057 | 0.883 |
| PCC | 0.476 | 0.195 | 0.646 | 0.060 | -0.279 | 0.467 | -0.110 | 0.7774 |
| AM | 0.548 | 0.127 | 0.595 | 0.091 | 0.093 | 0.812 | 0.191 | 0.623 |
| CN | 0.379 | 0.315 | 0.686 | ***0.041*** | 0.254 | 0.509 | 0.222 | 0.566 |
| GP | 0.531 | 0.141 | 0.249 | 0.519 | 0.045 | 0.908 | 0.415 | 0.267 |
| PN | 0.617 | 0.077 | 0.185 | 0.634 | 0.057 | 0.884 | -0.119 | 0.761 |
| MB | 0.421 | 0.260 | 0.468 | 0.243 | 0.561 | 0.116 | 0.323 | 0.435 |
| MED | 0.536 | 0.171 | 0.221 | 0.598 | 0.077 | 0.855 | 0.023 | 0.957 |
| CB | 0.292 | 0.445 | -0.169 | 0.665 | -0.545 | 0.129 | 0.040 | 0.919 |
| FWM | -0.341 | 0.370 | -0.466 | 0.206 | -0.242 | 0.530 | -0.571 | 0.108 |
| SPC | 0.527 | 0.224 | 0.438 | 0.278 | -0.647 | 0.116 | -0.532 | 0.175 |

Correlations between HO-1 RNA or protein and PSD95 or synaptophysin protein in individual regions were analyzed by Pearson’s correlation. Bold and italicized text: p<0.05

**Supplementary Table 8** Associations between brain HO-1 and synaptic markers in HIV-negative individuals

|  | **PSD95 protein vs. HO-1 RNA** | | **PSD95 protein vs. HO-1 protein** | | **Synaptophysin protein vs. HO-1 RNA** | | **Synaptophysin protein vs. HO-1 protein** | |
| --- | --- | --- | --- | --- | --- | --- | --- | --- |
| **Region** | **Pearson r** | **p-value** | **Pearson r** | **p-value** | **Pearson r** | **p-value** | **Pearson r** | **p-value** |
| FC | -0.184 | 0.727 | 0.003 | 0.995 | 0.215 | 0.682 | 0.791 | ***0.034*** |
| TC | -0.431 | 0.469 | 0.546 | 0.205 | -0.295 | 0.630 | 0.836 | ***0.019*** |
| OC | 0.717 | 0.070 | 0.753 | 0.051 | 0.057 | 0.903 | 0.381 | 0.399 |
| MC | 0.025 | 0.958 | 0.037 | 0.937 | 0.078 | 0.867 | 0.065 | 0.889 |
| SC | -0.490 | 0.324 | -0.209 | 0.653 | 0.489 | 0.325 | 0.130 | 0.781 |
| ACC | -0.038 | 0.936 | -0.100 | 0.831 | 0.205 | 0.660 | -0.266 | 0.564 |
| PCC | 0.213 | 0.647 | -0.149 | 0.749 | -0.140 | 0.766 | 0.458 | 0.3018 |
| AM | 0.286 | 0.534 | 0.056 | 0.905 | 0.286 | 0.535 | 0.770 | ***0.043*** |
| CN | -0.057 | 0.903 | -0.493 | 0.261 | -0.400 | 0.374 | -0.700 | 0.080 |
| GP | -0.019 | 0.969 | 0.514 | 0.238 | -0.307 | 0.503 | 0.589 | 0.164 |
| PN | 0.666 | 0.103 | 0.093 | 0.843 | 0.528 | 0.223 | 0.469 | 0.289 |
| MB | -0.055 | 0.907 | 0.300 | 0.513 | -0.170 | 0.716 | 0.503 | 0.250 |
| MED | -0.552 | 0.199 | 0.537 | 0.214 | -0.260 | 0.573 | 0.797 | ***0.032*** |
| CB | 0.339 | 0.511 | -0.423 | 0.344 | -0.409 | 0.421 | 0.360 | 0.428 |
| FWM | 0.133 | 0.776 | 0.756 | ***0.049*** | 0.178 | 0.702 | 0.639 | 0.123 |
| SPC | -0.647 | 0.238 | -0.246 | 0.595 | -0.829 | 0.083 | 0.248 | 0.592 |

Correlations between HO-1 RNA or protein and PSD95 or synaptophysin protein in individual regions were analyzed by Pearson’s correlation. Bold and italicized text: p<0.05
